# A Smart Fluid‐Hydraulic System Based on MRF Valve Control and Its Application in Flexible Grasping

**DOI:** 10.1002/advs.76435

**Published:** 2026-07-03

**Authors:** Linfeng Huo, Hui Ji, Ruidong Hong, Songlin Nie, Fanglong Yin, Zhonghai Ma

**Affiliations:** ^1^ College of Mechanical & Energy Engineering Beijing University of Technology Beijing China

**Keywords:** flexible manipulator, magnetorheological fluid, smart fluid‐hydraulic systems, soft actuator, variable stiffness

## Abstract

This study proposes a smart fluid hydraulic system based on magnetorheological fluid (MRF) valve control for flexible gripping. An MRF micro‐servo valve (MRF‐MSV) that integrates an excitation coil and micro‐textured damping channels has been designed. Through experiments and simulations based on the Bingham model, the hydraulic resistance characteristics of the valve under different currents are analyzed, which confirms the enhancing effect of the micro‐textured channels on hydraulic resistance. Furthermore, experiments demonstrate that at an excitation current of 3 A, the valve body's resistance pressure reaches 1191.4 kPa with a micro‐textured height of 0.2 mm, representing an 81.9% increase compared to the valve without micro‐textured channels (655 kPa). Integrating the MRF‐MSV with a soft actuator enables dynamic control of bending and grasping motions, with a maximum bending angle of 180°. Then, an MRF variable‐stiffness soft actuator (MRF‐VSSA) is developed. When a current of 1.2 A is applied to the variable‐stiffness module, the fingertip force increases from 2.69 to 5.05 N, showing an 87.7% increase. Finally, an MRF‐based smart fluid‐hydraulic system is constructed, and a magnetically driven manipulator is developed. Experiments indicate that this manipulator can execute complex hand movements and stably grasp objects of various shapes without causing damage.

## Graphical abstract

This study introduces a smart fluid hydraulic system using MRF valve control for flexible gripping. An MRF‐MSV with an integrated excitation coil and micro‐textured damping channels is designed and validated; micro‐texturing increases hydraulic resistance. Coupled with a soft actuator, the MRF‐MSV enables dynamic bending and grasping. A full MRF‐based smart hydraulic manipulator achieves stable, non‐damaging grasping of diverse objects.

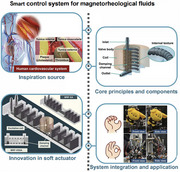

## 1 Introduction

In complex operational scenarios that involve a wide range of objects and shapes, the traditional rigid fixture, typically made of metal or engineering plastics, has a high bearing capacity. However, its versatility and adaptability are inadequate, making it difficult to meet the requirements for the efficient processing of diverse objects [1, 2, 3, 4]. When dealing with deformable objects or precision components, specialized custom designs are frequently necessary [5, 6, 7]. Moreover, to achieve damage‐free handling, rigid grippers must rely on precise force sensing as a prerequisite and integrate high‐precision sensors, flexible drive units, and robust control systems [8, 9, 10].

Innovations in soft robotics technology have created new possibilities for human‐machine interaction and precision manipulation. Flexible systems founded on biomimetic principles have exhibited substantial advantages in domains such as grasping, motion control, environmental perception, rehabilitation assistance, and minimally invasive medicine [11, 12]. Among these, soft actuators fabricated from flexible materials, due to their high flexibility and energy‐dissipating characteristics, can adaptively conform to fragile objects and irregular surfaces [13, 14, 15, 16, 17, 18]. This technology showcases positive performance in multiple aspects, including facilitating safer human‐robot collaboration, enabling lightweight structural design, possessing multi‐degree‐of‐freedom motion capabilities, simplifying control algorithms, having a certain degree of impact robustness, allowing for modular rapid prototyping, and incurring lower manufacturing costs [19, 20]. Consequently, it establishes a core competitive edge in smart manufacturing [21], medical rehabilitation [22, 23, 24, 25], and specialized operations [26, 27, 28]. Among various soft drive methods, hydraulic drive systems surpass smart material‐driven robots in terms of safety, user‐friendliness, environmental adaptability, and functional diversity. However, they still encounter challenges such as system complexity, energy supply, and control latency [29, 30, 31].

**FIGURE 1:**
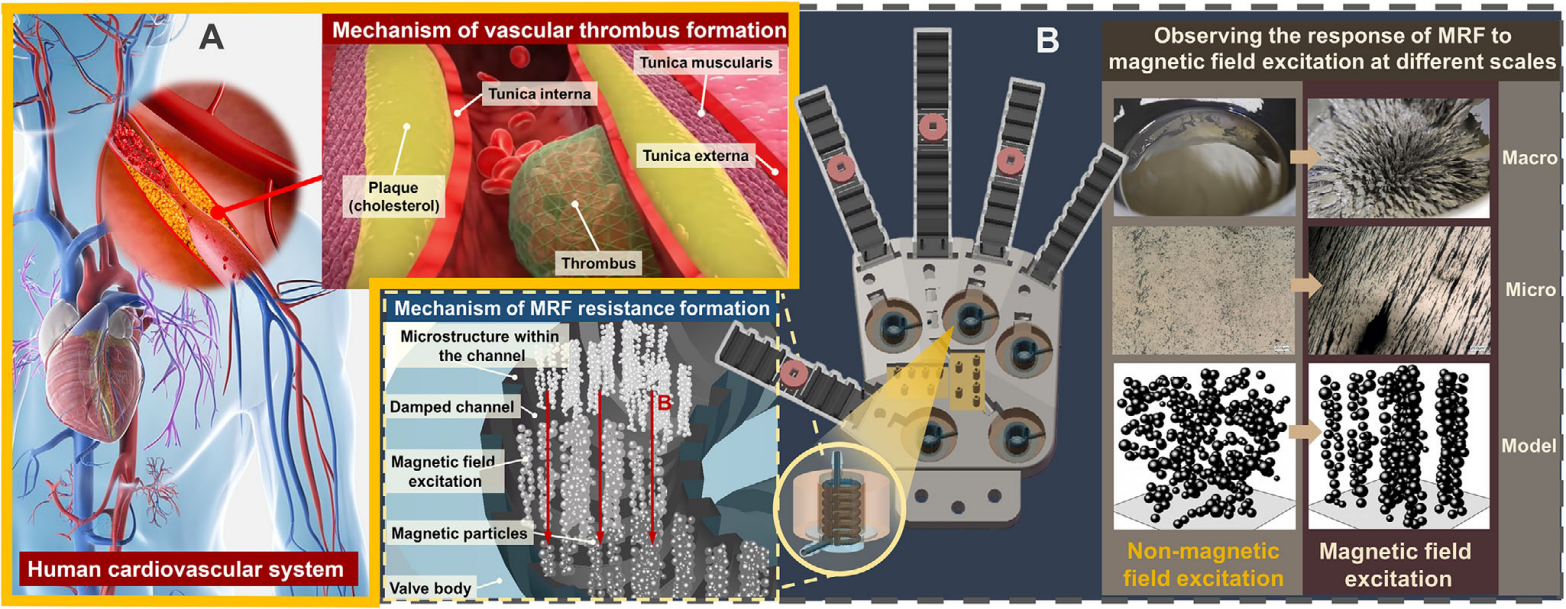
Magnetic‐controlled mechanism and bionic design of flexible manipulators based on magnetorheological fluid. (A) Bionic design inspiration derived from the process of vascular thrombus formation in the human cardiovascular system. (B) Observation of magnetorheological fluid behavior under magnetic field excitation at different scales; this phenomenon is applied to the MRF‐MSV mechanism of the manipulator to achieve magnetic‐controlled actuation.

The human cardiovascular system is essentially a sophisticated biological hydraulic system. Vascular embolism pertains to the formation of a pressure‐driven obstruction by platelets in reaction to specific stimuli, leading to ischemia in distal tissues [32, 33]. As depicted in Figure 1A, vascular embolism offers distinctive biomimetic inspiration for pressure control in soft robots: if the rheological characteristics of the working fluid can be locally and reversibly modified to create a “fluid embolism,” mechanical valves with moving components can be eliminated. In hydraulically actuated soft robots, system pressure is generally attained by adjusting the orifice aperture of a servo valve, and its dynamic performance is contingent upon the precision of the actuator [34, 35, 36]. Emerging soft valves impede flow by twisting the tubing to form local bends [37, 38] or by radially compressing the tubing to continuously regulate flow resistance [39]. The former can only achieve on‐off control, whereas the latter is intricate to manufacture and has a restricted range of sealed pressure regulation.

Magnetorheological fluid (MRF) is a smart fluid formed by suspending nano‐ or micron‐sized magnetic particles in a carrier liquid. Under the influence of an external magnetic field, its viscosity and yield stress undergo continuous, reversible changes on the order of milliseconds [40, 41], and it has been widely applied in semi‐active automotive suspension systems, pipeline vibration control systems and similar applications [42, 43, 44, 45]. In recent years, there has been a growing body of research on the integration of magnetorheological fluids (MRFs) into robotic systems. By utilizing the shear yield stress of MRFs, controllable enveloping grasping has been achieved, thereby adapting to the geometric morphology of the object being grasped [46, 47, 48, 49]. Furthermore, the addition of non‐magnetic particles to the sac‐like structure of planar grippers can enhance gripping force [50]. Mixing MRF with scaffold materials forms hybrid stuffed structures with enhanced stiffness, and the stiffness can be actively adjusted via electromagnets [51]. Combining MRF with wire‐driven robots enables the creation of laparoscopic surgical instruments with variable‐stiffness joints [52] and non‐motorized hand exoskeletons with enhanced grip strength [53]. Integrating MRF into flexible robotic systems allows for binary on‐off flow control via magnets, thereby enabling selective actuator actuation [54, 55]. In the field of microvalves, previous research has utilized MRF droplets as switching elements to control the opening and closing of microchannels [56] or has employed temperature‐controlled magnetic circuits to achieve proportional pressure regulation in micro‐MR valves [57]. Nevertheless, the primary disadvantages of magnetic microvalves are their insufficient pressure resistance and susceptibility to leakage [58]. Likewise, although microvalves based on electrorheological fluids have achieved comparable control performance, their response speed and pressure resistance are also restricted [59]. Despite these advances, existing MRF‐based robotic systems share common limitations: they primarily utilize the shear yield stress of MRF to achieve binary on‐off actuation or simple damping. Damping channels typically feature smooth walls, requiring strong magnetic fields or long flow paths to achieve sufficient flow resistance. There are a few reports of work integrating continuous proportional flow control with active stiffness regulation within the same fluidic system.

This study demonstrates that when MRF flows through a damped channel under an applied magnetic field, its viscosity undergoes continuous and reversible changes, exhibiting solid‐like behavioral characteristics. By introducing rectangular ring micro‐textured structures into the damped channel, the resistance to the flow of high‐viscosity MRF under limited magnetic field strength was significantly enhanced, thereby effectively simulating the hemodynamic occlusion effects caused by vascular embolism in the human body. Based on MRF's rheological properties, magnetic control resistance tests confirmed its potential for soft robotics actuation. Initially, a micro servo valve (MRF‐MSV) integrating a meandering‐structured damping channel was designed, as depicted in Figure 1B. This valve employs MRF as its working medium and is magnetically excited by an external excitation coil. Through simulation analysis, the mechanism by which the micro‐texturing of the damping channel affects the viscosity changes of MRF was investigated. By integrating the MRF‐MSV with magnets, dynamic control over the deformation and grasping motions of the flexible gripper and soft trunk actuator was successfully realized. After replacing the magnets with controllable excitation coils, the hydraulic resistance characteristics of the MRF‐MSV were measured under various input current conditions. Moreover, the developed MRF‐MSV was connected to a soft actuator (MRF‐SA) to examine the impact of current regulation on the deformation behavior of the soft actuator. Subsequently, a similar damping channel structure was incorporated into a soft actuator to propose an MRF‐based variable stiffness soft actuator (MRF‐VSSA), and an in‐depth analysis was conducted on its deformation behavior and fingertip force output characteristics.

The MRF‐MSV designed in this study demonstrated an 81.9% increase in pressure resistance at a current of 3 A, with the soft actuator achieving a maximum bending angle of 180°; the variable stiffness module increased the fingertip force by 87.7%. Building on this, by integrating valve control systems with flexible drive technology, a smart hydraulic system based on magnetorheological fluid transmission and control was established, and a magnetically driven smart fluid manipulator was developed. Experiments validated the manipulator's performance in executing complex gestures, performing non‐destructive grasping of objects with complex shapes, and maintaining a stable grip on target objects.

## 2 Results

### 2.1 The Magnetically Controlled Resistance of MRF

**FIGURE 2:**
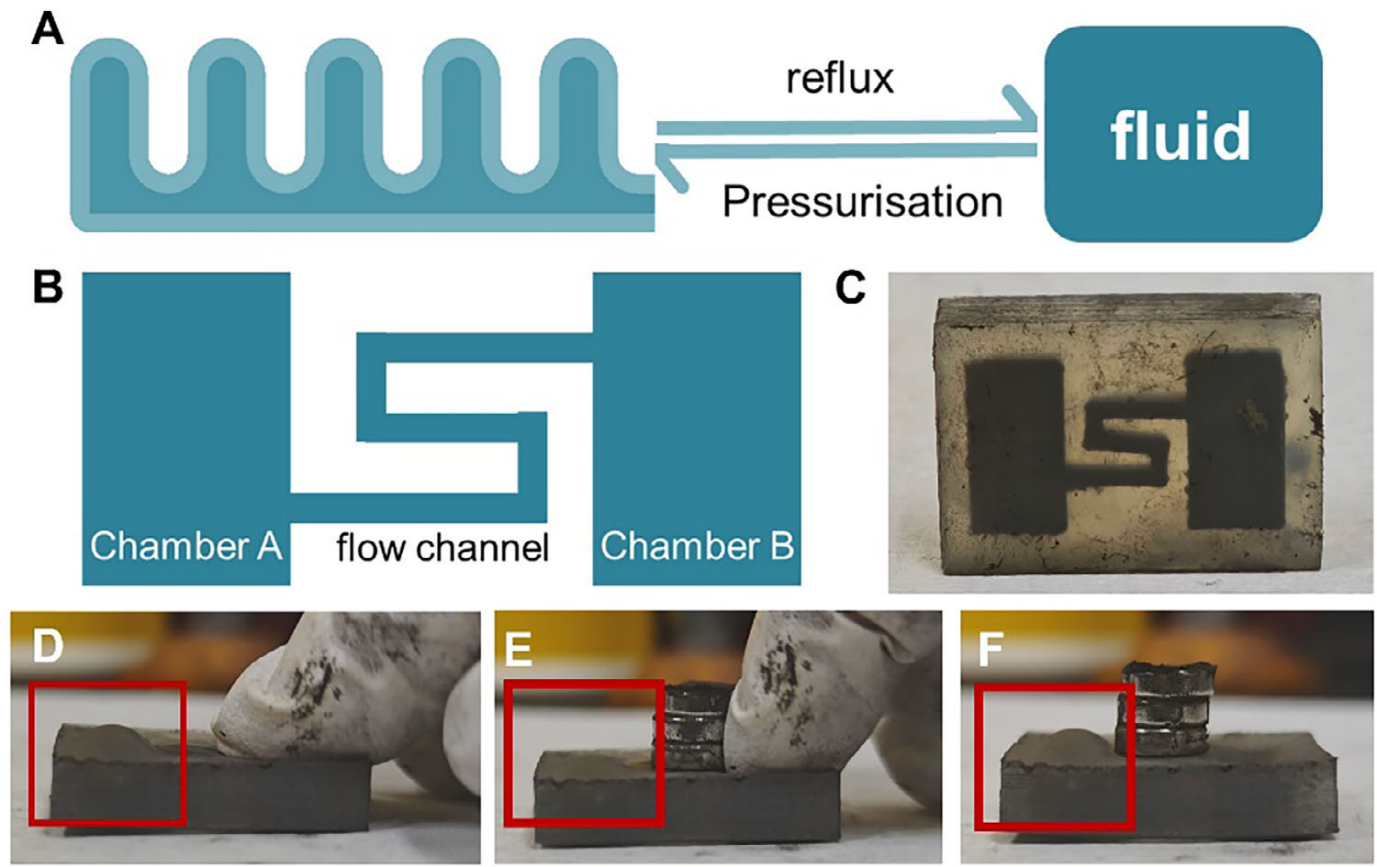
The principle of magnetically controlled resistance in MRF. (A) Operating principle of a fluid pressure‐driven soft actuator. (B) Operating principle and internal structure of a magnetic control resistance demonstration model. (C) Silicone‐molded magnetic control resistance demonstration model. (D) Applying pressure to the chamber without a magnetic field induces the magnetorheological fluid to flow into the other chamber, causing its inflation. (E) Applying pressure to the chamber with an applied magnetic field enables the flow channel to effectively inhibit the flow of the magnetorheological fluid. (F) Magnetic field control can achieve the blocking of the backflow of the magnetorheological fluid within the inflated chamber.

Fluid pressure‐driven soft actuators operate by pumping fluid media stored within a fluid tank into a sealed chamber within the actuator via an external pressure source. Valves enable precise control and locking of the chamber pressure. During this process, expansion of the chamber volume or changes in its shape drive the actuator to produce a predetermined deformation or action. Upon task completion, the valve opens, allowing the pressurized fluid within the chamber to flow back into the fluid tank under the elastic restoring force of the flexible material itself. The system thereby resets, forming a recyclable drive closed loop, as illustrated in Figure 2A.

Magnetorheological fluid is a quasi‐non‐Newtonian fluid whose apparent viscosity can be modulated by an external magnetic field. To investigate its applicability in soft actuator systems, this study designed the verification structure depicted in Figure 2B. This structure comprises a magnetically controlled impedance demonstration model fabricated via silicone molding, containing two chambers (A and B) interconnected by a tortuous flow channel, with the fabrication process shown in Figure S3. Magnetorheological fluid was injected into the model, as depicted in Figure 2C. The validation experiment proceeded as follows: as shown in Figure 2D, pressing chamber B caused the magnetorheological fluid to flow through the channel into chamber A under pressure, resulting in the inflation of chamber A. Upon releasing the pressure, chamber A returned to its original state due to the material's elastic properties, and the fluid flowed back into chamber B. As shown in Figure 2E, after applying a magnetic field above the flow channel, chamber B is pressed again. At this point, the magnetorheological fluid significantly thickens due to the magnetorheological effect, resisting flow and preventing chamber A from inflating. Applying the magnetic field thus inhibits the flow of the magnetorheological fluid. As shown in Figure 2F, chamber B is first compressed to create an indentation. After chamber A inflates, a magnetic field is applied above the flow channel, and the pressure on chamber B is released. At this point, the high‐pressure fluid within chamber A is confined by the magnetorheological fluid exhibiting rheological effects within the flow channel, thereby maintaining its shape. Upon magnetic field removal, the fluid regains fluidity and flows back into chamber B under elastic restoring force, restoring chamber A to its original state while filling the indentation in chamber B. The magnetic field thus locks the soft actuator's shape. The above validation experiments demonstrate that when using magnetorheological fluid as the driving medium, its flow characteristics can be controlled via an external magnetic field. This enables non‐contact, reversible regulation of the soft actuator's action drive and state locking, functioning equivalently to a switch valve and a pressure‐holding valve (Movie S1 demonstrates the testing of the magnetically controlled impedance demonstration model).

### 2.2 MRF‐MSV Based on Magnetorheological Fluid

By applying an external magnetic field, the flow of MRF can be switched on and off, thereby enabling switch‐driven expansion and shape‐locking of flexible chambers. The core of this magnetically controlled impedance capability lies in the matching of the magnetic field with the damping flow channels.

The flow path of the MRF in the MRF‐MSV is shown in Figure S2B. The design principle of MRF‐MSV involves applying a magnetic field perpendicular to the flow direction as magnetorheological fluid passes through a long conduit. The operational model of the MRF in this study is a flow‐type model, as shown in Figure S2C. A rheological effect is induced, transforming the fluid from a liquid to a semi‐solid state, thereby creating resistance against the pressurized fluid. Consequently, the valve body structure design prioritizes optimizing the spatial positioning of the excitation coil's magnetic field under current excitation and streamlining the dimensional layout of the valve assembly. The excitation coil is fitted externally around the valve body, whilst the damping channel within the valve body adopts a serpentine converging configuration. The excitation coil generates a magnetic field perpendicular to the direction of MRF flow, forming the relatively simple structure depicted in Figure S4. The advantage of positioning the excitation coil outside the valve body lies in the fact that the magnetic field is perpendicular to the direction of MRF flow (flow pattern), which leads to high pressure regulation efficiency. Moreover, the coil does not need to be embedded in the soft actuator, thus avoiding any impact on the flexibility of the soft material. Additionally, it also facilitates heat dissipation and maintenance.

**FIGURE 3:**
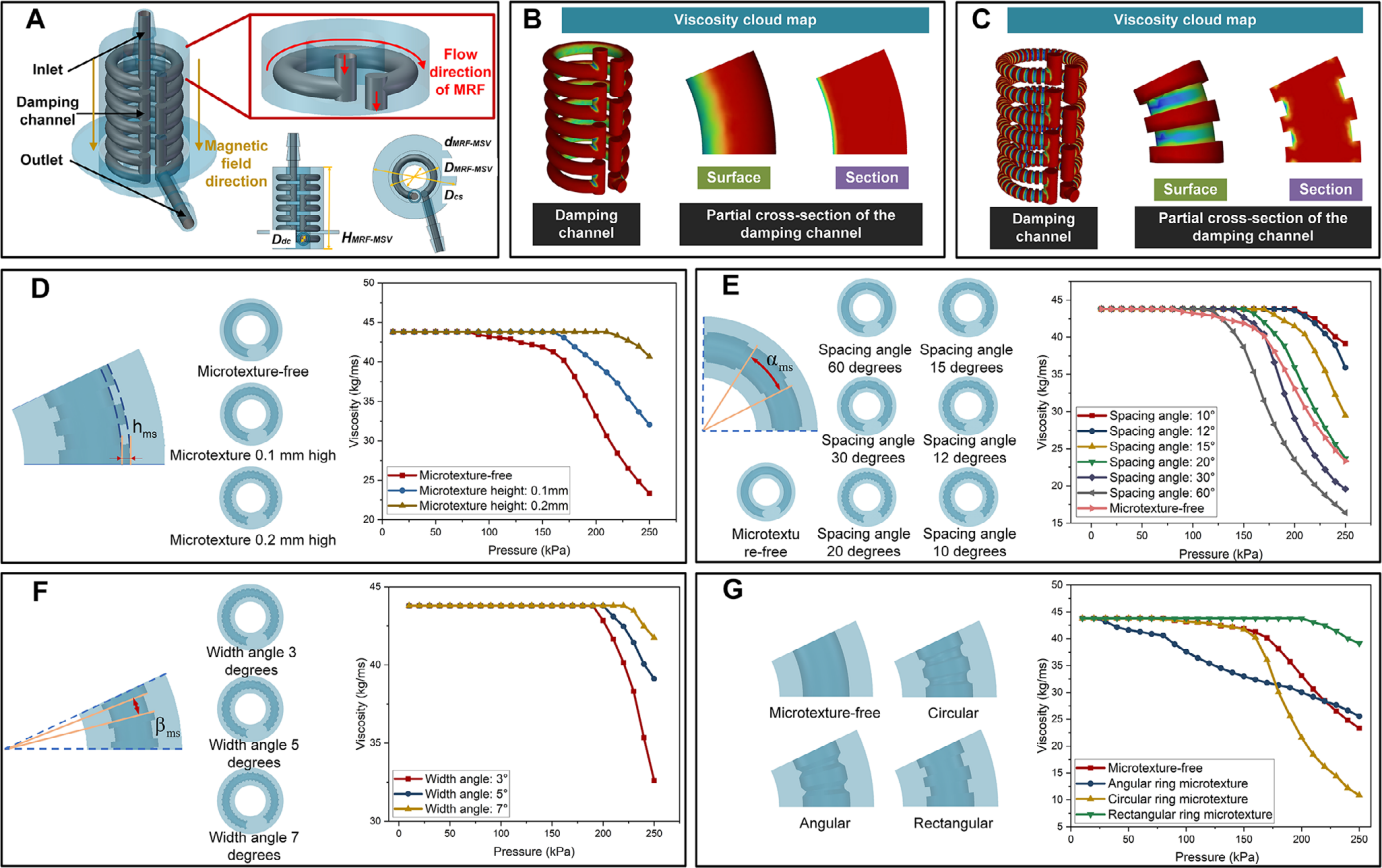
Study on the relationship between MRF‐MSV structural parameters and resistance capability. (A) The fluid domain of MRF‐MSV. (B) The influence of smooth damping channels on the viscosity of magnetorheological fluid in MRF‐MSV. (C) The influence of micro‐textures in damping channels on the viscosity of magnetorheological fluid in MRF‐MSV. (D) The effect of micro‐texture heights on MRF viscosity under different pressures. (E) The influence of micro‐texture spacing angle on MRF viscosity under different pressures. (F) The influence of micro‐texture width angle on MRF viscosity under different pressures. (G) The influence of various micro‐texture shapes on MRF viscosity under different pressures.

In Figure 3A, the valve body of the MRF‐MSV comprises the inlet and outlet ports along with the internal damping flow channel. The valve body height *H_MRF‐MSV_* is 24 mm, the valve body internal diameter *d_MRF‐MSV_* is 7 mm, the valve body external diameter *D_MRF‐MSV_* is 15 mm, and the coil support external diameter *D_cs_* is 24 mm, the damping channel exhibits a circular cross‐section with an internal diameter *D_dc_* is 2 mm.

As shown in Figure 3, to enhance the resistance effect on MRF within a limited space and damping channel length, a stronger magnetic field strength is needed to be applied as the fluid flows through. However, this approach imposes higher demands on the efficiency of the excitation coil. In light of this, micro‐textures were incorporated into the damping channel to enhance the resistance effect. The influence of key structural parameters such as spacing angle *α_ms_*, width angle *β_ms_*, structure shape, and height *h_ms_* on the resistance characteristics is investigated. ANSYS Fluent simulation is conducted to examine changes in fluid viscosity and determine the failure pressure of the MRF‐MSV. Given the identical damping channel configuration and consistent channel length across all layers of the MRF‐MSV body, the relationship between texture and flow resistance characteristics can be effectively investigated using a single‐layer damping channel as the representative model. In the MRF‐MSV, the MRF enters through the inlet, flows through the single‐layer damping channel in the direction of the red arrow into the next layer, and exits through the outlet, as shown in Figure 3A. Furthermore, the issue of excitation coil heating is neglected due to the relatively short operational duration of the MRF‐MSV in the current simulation.

The excitation coil is energized to generate a magnetic field (Figure 3B,C). When pressurized magnetorheological fluid flows through the MRF‐MSV, it undergoes a rheological effect and attains an increased viscosity. Under the combined influence of fluid pressure and an external magnetic field, the viscosity evolution of MRF within a micro‐texture‐free damping channel exhibits a layered annular arrangement as the fluid pressure increases, ultimately forming a distribution characterized by high viscosity at the center and low viscosity at the periphery, as shown in Table S1. In contrast, the viscosity variation of MRF within the micro‐textured damping channel is more complex. As indicated in Table S2, viscosity reduction first occurs at the junctions between adjacent micro‐texture grooves, whilst MRF within the grooves maintains a high‐viscosity state. With increasing fluid pressure, these low‐viscosity regions gradually expand and merge. This ultimately isolates the high‐viscosity MRF within the micro‐texture grooves from the high‐viscosity region at the channel center, forming a distinctive distribution pattern: high viscosity in the central damping channel and micro‐texture grooves, and low viscosity at the groove junctions. By disrupting the continuous low‐viscosity path along the channel edges, the damping channel with a micro‐texture configuration hinders the pressure‐driven flow. As a result, it enhances the magnetorheological fluid's (MRF) pressure resistance by 162.5% under a finite magnetic field. This enhancement is based on a compressive strength of 80 kPa for the unstructured material in the simulation model and a compressive strength of 210 kPa for the material with a micro‐texture height of 0.2 mm. Consequently, it significantly improves the overall performance of the MRF‐MSV.

Subsequently, a simulation analysis was performed to analyze the viscosity behavior of MRF as it flows under varying pressures into a damping channel with distinct micro‐texture structures. Simulation studies of different micro‐texture heights *h_ms_* indicate that *h_ms_* can effectively enhance the resistance pressure, as shown in Figure 3D. Within the MRF‐MSV damping channel, the MRF undergoes a rheological transformation under the influence of an excitation magnetic field, transforming into a high‐viscosity state. As micro‐texture height increases, the pressure required to induce viscosity changes in the high‐viscosity MRF within the damping channel also rises. The variation in viscosity is attributed to the fragmentation of magnetic particle chains from the high‐pressure MRF impact, which in turn disrupts the pressure‐resistance function of the high‐viscosity MRF, causing the MRF to flow. This critical pressure value altering the MRF viscosity, represents the maximum operating pressure for MRF‐MSV. Regarding the pressure required to trigger viscosity changes, a 0.2 mm structure height can improve pressure resistance capability by approximately 162.5%.

Simulation studies of micro‐texture spacing angle *α_ms_* demonstrate that the damping channel exhibits significantly lower resistance effects on the magnetorheological fluid compared to a damping channel without micro‐textures when *α_ms_* surpasses 20°, as shown in Figure 3E. Conversely, it is only when the micro‐texture spacing angle is below 20° that the micro‐textures within the damping channel exhibit resistance effects on the magnetorheological fluid. The smaller the micro‐texture spacing angle *α_ms_*, the more effective the resistance effect produced. Therefore, *α_ms_* is selected as 10°, serving as the textural parameter for the damping channel.

In simulation studies of micro‐texture width angle *β_ms_*, as shown in Figure 3F, the larger the width angle of micro‐textures in the damping channel, the better the resistance effect on the magnetorheological fluid. However, selecting the maximum micro‐texture width angle results in relatively smaller micro‐texture grooves, given that the total length of the damping channel remains unchanged. Specifically, when *β_ms_* is 7°, the wide angle of the micro‐texture groove is merely 3°, accompanied by a minimum groove spacing of around 0.235 mm. Drawing from experience in photopolymerization 3D printing, this scenario poses challenges in thoroughly eliminating or drying residual resin from the grooves during post‐processing. Following secondary curing, micro‐texture grooves are prone to clogging. Consequently, a suboptimal structural parameter is selected for manufacturing to ensure production feasibility, namely, selecting a micro‐texture width angle with 5° as the texture parameter for the damping channel.

In a simulation examining various micro‐texture shapes, as depicted in Figure 3G, it is observed that both angular ring and circular ring micro‐textures weaken the damping channel's resistance effect on the magnetorheological fluid, causing viscosity changes at low pressures within the damping channel. In contrast, rectangular ring micro‐textures effectively enhance the damping channel's resistance effect on the magnetorheological fluid, making this shape the preferred choice.

Simulation results show that a 0.2 mm rectangular ring micro‐texture can increase the pressure resistance of the MRF‐MSV by 162.5%. This indicates that, without increasing coil power, a well‐designed micro‐texture can significantly enhance the valve's hydraulic resistance characteristics, thereby providing a more efficient magnetically controlled fluid locking mechanism for soft robots.

### 2.3 Locking Control of Soft Actuator Action

This study experimentally verified the feasibility of MRF‐MSV in driving and locking soft actuators. Simulation results indicate that MRF‐MSV exhibits significant flow resistance to MRF under high pressure. In the experiment, an injector pressurized the MRF, which flowed through the MRF‐MSV before being injected into the soft actuator. An externally applied magnetic field controlled the fluid flow on/off state.

**FIGURE 4:**
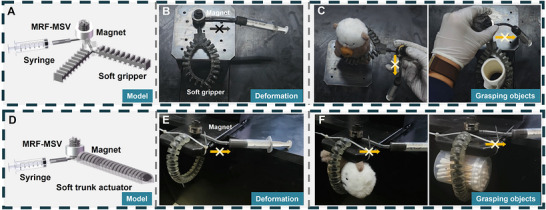
Action control of magnetically controlled soft actuators. (A) Schematic diagram of the magnetically controlled flexible gripper structure. (B) The gripper is undergoing closure deformation under magnetic field control. (C) Stable grasping performance of the flexible gripper on a plush toy and a toilet roll. (D) Schematic diagram of the magnetically controlled soft trunk actuator. (E) Curling deformation process of soft trunk actuator under magnetic field regulation. (F) Demonstration of soft trunk actuator's coiling grasp of plush toy and cotton bud box.

The flexible gripper undergoes a closing deformation under fluid pressure (Figure 4A–C). By encircling the MRF‐MSV with a ring magnet, the backflow of high‐pressure magnetorheological fluid is effectively blocked, thereby maintaining the gripper in a pinching state. This successfully achieves the grasping and retention of a plush toy and a roll of toilet paper. The soft trunk actuator undergoes curling deformation under pressure (Figure 4D–F). Similarly, deformation locking is achieved by externally mounting magnets on the valve, enabling the winding and capture of a plush toy and a cotton bud box. (Movie S2 demonstrates the action control test of magnetically controlled soft actuators.)

Experiments have demonstrated that the MRF‐MSV can switch the flow state of the MRF via an external magnetic field, enabling the deformation and position locking of flexible grippers and soft trunk actuators, and successfully grasping objects of various shapes. This indicates that the magnetically controlled resistance characteristics of the MRF can be effectively applied to the fluid control and state maintenance of soft actuators.

### 2.4 Variable Magnetic Field Control Resistance Capability

To enhance the controllability of magnetically controlled soft robots, fixed magnets were upgraded to electromagnetic coils, enabling dynamic regulation of the magnetic field surrounding the MRF‐MSV. This not only allows for continuous adjustment of its resistive capacity but also facilitates modulation of the internal drive pressure within the soft actuator. Subsequent experiments will further test the resistive performance under variable magnetic fields and validate the practical efficacy of the micro‐texture design in improving magnetically controlled resistive characteristics.

**FIGURE 5:**
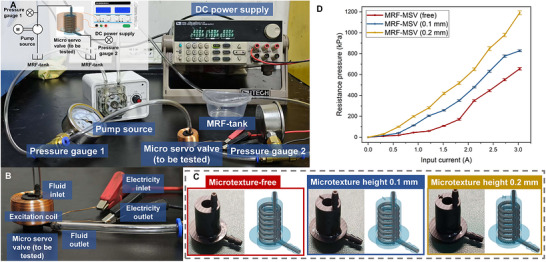
Pressure resistance characteristic test of MRF‐MSV. (A) Hydraulic test system for MRF‐MSV. (B) The electrical interface and hydraulic line connections of MRF‐MSV. (C) Physical specimens and models of MRF‐MSV test samples with different micro‐texture heights. (D) Resistance characteristics of MRF‐MSV with different micro‐texture heights under varying input currents. The error bars represent the standard deviation of the mean from five experimental trials.

To validate the resistance characteristics of the rectangular ring micro‐textures, a hydraulic test system was subsequently established, as shown in Figure 5A, to evaluate the pressure resistance properties of MRF‐MSV. A magnetorheological fluid was delivered into the pipeline of the test system via a peristaltic pump. Pressure gauge 1 measured the pre‐valve resistance pressure, and pressure gauge 2 measured the hydraulic system line pressure. By adjusting the input current to the excitation coil in the MRF‐MSV, different magnetic field strengths were generated, controlling the viscosity of the magnetorheological fluid within the valve to produce varying resistance pressures. The electrical interface and hydraulic line connections of the MRF‐MSV are shown in Figure 5B.

As shown in Figure 5C, three types of MRF‐MSV with different rectangular ring micro‐texture heights were fabricated via photopolymerization‐3D printing. Their pressure resistance characteristic under varying input currents was investigated using a hydraulic test system. Under the same experimental conditions, each MRF‐MSV was tested five times, and the mean and standard deviation were calculated. Figure 5D demonstrates that designing micro‐texture grooves on the damping channel within the valve significantly enhances pressure resistance capability. When the input current was 3 A, the average resistance pressure of the MRF‐MSV without micro‐textures, with a micro‐texture height of 0.1 mm, and with a micro‐texture height of 0.2 mm were 655, 827.4, and 1191.4 kPa, respectively. When the micro‐texture height is 0.2 mm, the pressure resistance capacity increases by 81.9% compared to the configuration without micro‐texture. To improve energy efficiency, reduce coil heating, and achieve higher resistance pressure under the same current conditions—or maintain target resistance pressure at reduced control currents—subsequent research focused primarily on the MRF‐MSV with a rectangular ring micro‐textured damping channel with a 0.2 mm micro‐texture height.

The results above indicate that rectangular ring micro‐textures significantly enhance the resistance pressure of the MRF‐MSV. At a current of 3 A, the resistance pressure value for a micro‐texture height of 0.2 mm was 81.9% higher than that of the unstructured version. (In the simulation, the compressive strength increase (162.5%) at a micro‐texture height of 0.2 mm was significantly higher than the experimental value (81.9%). This may be attributed to the non‐uniform distribution of the magnetic field generated by the actual excitation coils, whereas the simulation assumed a uniform magnetic field). This implies that adding micro‐textures allows for a higher fluid locking pressure to be achieved at the same excitation power consumption, thereby providing a wider range of force control for soft actuators.

As presented in Table 1, in comparison with other smart fluidic microvalves, the MRF‐MSV developed in this study is characterized by a considerably broader pressure regulation range, allows for continuous proportional control, and demonstrates rapid electromagnetic response characteristics. In contrast to electro‐rheological valves [59], which require 1.5 kV, MRF‐MSV utilizes a MRF as its flow medium and operates at a voltage of just 4 V. This characteristic makes it safer and easier to integrate into soft robots. Its compact design also enables seamless integration in space and performance‐constrained applications.

**TABLE 1:** Summary and comparison of the performance of smart fluid microvalves.

Reference	Drive type	Fluid	Control type	External field	Pressure adjustment range (kPa)	Dimension (mm)
This study	Excitation coil	MRF‐15D	Proportional control	100 mT (Magnetic field)	60–1191.4	φ31.5 × 24
[54, 55]	Permanent magnet	Homemade Water‐Based MRF	Two‐position switch	33 mT (Magnetic field)	0–4	15 × 15 × 5 (Estimating scale)
[56]	Permanent magnet array	Oil‐based MRF	Two‐position switch	—	0–0.6	50 × 7 × 10
[57]	Temperature‐controlled magnetic field	MRF‐132LD	Proportional control	40–65 mT (Magnetic field)	52–131	10 × 10 × 10
[59]	Electric Field	Electrorheological fluid	Proportional control	1.5 KV (electric field)	0–300	22 × 22 × 22

### 2.5 Application of Damping Channel in MRF‐SA

Previous studies have confirmed that adjusting the input current to the MRF‐MSV enables precise generation of specific resistance pressure within the magnetorheological fluid hydraulic system, with the resulting pressure being directly proportional to the applied current. This method of directly modulating fluid viscosity to create resistance through current‐induced magnetic excitation can substantially simplify the architecture of hydraulic‐driven soft actuators, thereby eliminating redundant structural components within the system.

In this study, we employed magnetorheological fluid as the transmission medium and integrated an MRF‐MSV as the core pressure regulation unit within the hydraulic circuit. Furthermore, we propose a design scheme that integrates the internal damping channel of MRF‐MSV with a soft actuator structure. This integrated structure achieves variable stiffness functionality within the soft actuator by leveraging its adjustable resistance characteristics.

**FIGURE 6:**
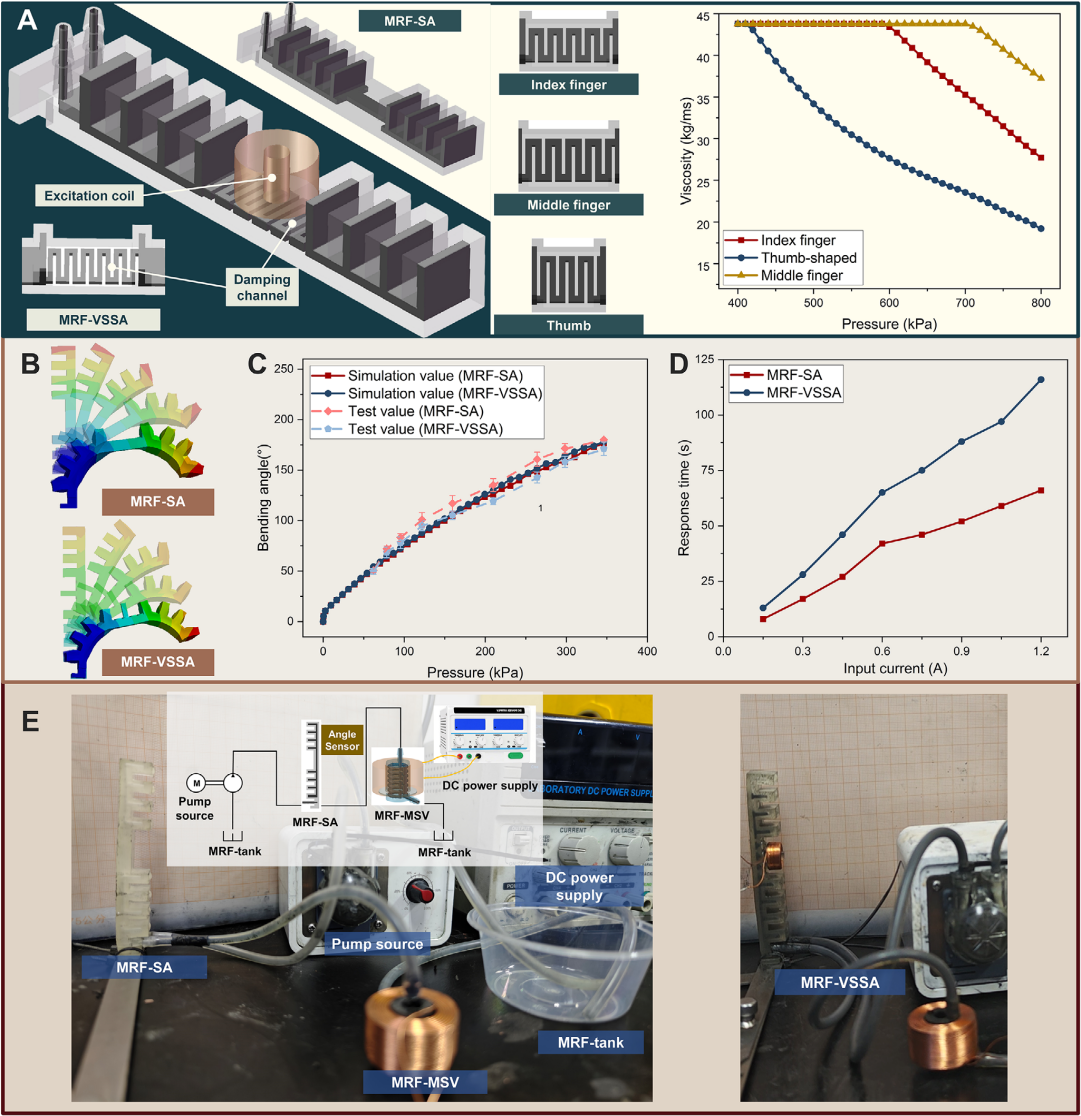
Deformation test for MRF‐SA and MRF‐VSSA based on MRF‐MSV. (A) Application of variable stiffness module on MRF‐SA and simulation of pressure resistance characteristics for different MRF‐VSSAs. (B) Bending deformation simulation of MRF‐SA and MRF‐VSSA. (C) Bending angle simulation curves for MRF‐SA and MRF‐VSSA, alongside experimental data of bending angles under varying input currents. The error bars represent the standard deviation of the mean from five experimental trials. (D) Relationship curves between bending deformation and response time for MRF‐SA and MRF‐VSSA under different input currents. (E) Hydraulic circuit for MRF‐SA and MRF‐VSSA bending deformation testing.

We modified a multiple‐finger soft manipulator—comprising actuators for the thumb, index, middle, and ring fingers (with the index and ring fingers sharing the same structure)—by adding variable stiffness modules, specifically dimensioned damping channels, into each soft actuator. The vacant section in the central area of the MRF‐SA has been fitted with variable stiffness modules. The centerline lengths of the damping channel for the thumb, index, and middle finger were 97, 109 mm, and 118 mm, respectively, with a cross‐sectional width and height of 2 mm each, as shown in Figure 6A. Pressure resistance simulations were subsequently conducted. Results indicate that longer damping channels elevate the pressure limit at which magnetorheological fluid exhibits viscosity changes under magnetic fields. Macro‐wise, longer channels demonstrate stronger resistance capabilities. All channel specifications demonstrated effective resistance performance within the actuator's operating pressure range (0–350 kPa), meeting the variable stiffness design requirement. Since all finger actuators share an identical design, the subsequent studies on the bending deformation and fingertip force of both MRF‐SA and MRF‐VSSA will use the index finger actuator as the representative case.

Standard dumbbell‐shaped tensile specimens were fabricated by photopolymerised 3D printing using F39T flexible photosensitive resin as the substrate. To obtain the hyperelastic mechanical parameters of this material, uniaxial tensile tests were conducted (Figure S6). Parameters for the Yeoh third‐order constitutive model were fitted based on experimental data, with specific values listed in Table S3. Finally, the material parameters were incorporated into finite element simulations. Figure 6B–D compares the response characteristics of the MRF‐SA and the MRF‐VSSA in simulation and experiment. Simulation results indicate that the bending deformation of MRF‐SA and MRF‐VSSA exhibits a high degree of consistency. Their bending deformation curves are virtually identical during the low‐pressure phase, with only minor deviations appearing as the pressure increases. This demonstrates that the variable stiffness module introduced in MRF‐VSSA does not significantly affect the overall bending deformation of the actuator. However, the incorporation of a variable stiffness module in MRF‐SA, in which the tortuous and narrow flow channels have a significant impact on the flow velocity of the high‐pressure magnetorheological fluid, considerably reduces the actuator's deformation response speed (In the experiments, the peristaltic pump flow rate was set at 25–50 mL/min). To validate actual performance, we constructed a magnetorheological fluid hydraulic experimental system (Figure 6E). The magnetorheological fluid resistance was controlled by adjusting the excitation coil current (0–3, 0.3 A increments) of the MRF‐MSV.

Based on the MRF‐MSV current‐resistance pressure calibration relationship, bending deformation data for both actuators under different resistance pressures were obtained. Under the same experimental conditions, each MRF‐MSV was tested five times, and the mean and standard deviation were calculated. The scatter plots dashed data in Figure 6C (from left to right) represent the deformation responses of both actuators under valve control as current increases. Specifically, the actual bending deformation of MRF‐SA was slightly higher than the simulated value; whereas for MRF‐VSSA, the experimental value in the low‐pressure zone was marginally higher than the simulated value, whilst in the high‐pressure zone it agreed well with the simulation results. The maximum average bending angle of the MRF‐SA was 180°, while that of the MRF‐VSSA was 171.2°. Notably, the experimental data reproduced the trend observed in the simulation: while the bending deformation of the MRF‐SA and MRF‐VSSA was closely matched at low pressure, the MRF‐SA exhibited minimally greater deformation as pressure increased. Although differences in deformation were observed between the two in the bending deformation experiments, their overall trends were highly similar. (At the maximum resistance pressure, the MRF‐SA achieved the maximum bending angle of 180° in all five tests.) This indicates that introducing variable stiffness modules into the central void region of MRF‐SA did not significantly impact the actuator's overall deformation capability, thereby laying the groundwork for subsequent research into modular variable stiffness systems.

**FIGURE 7:**
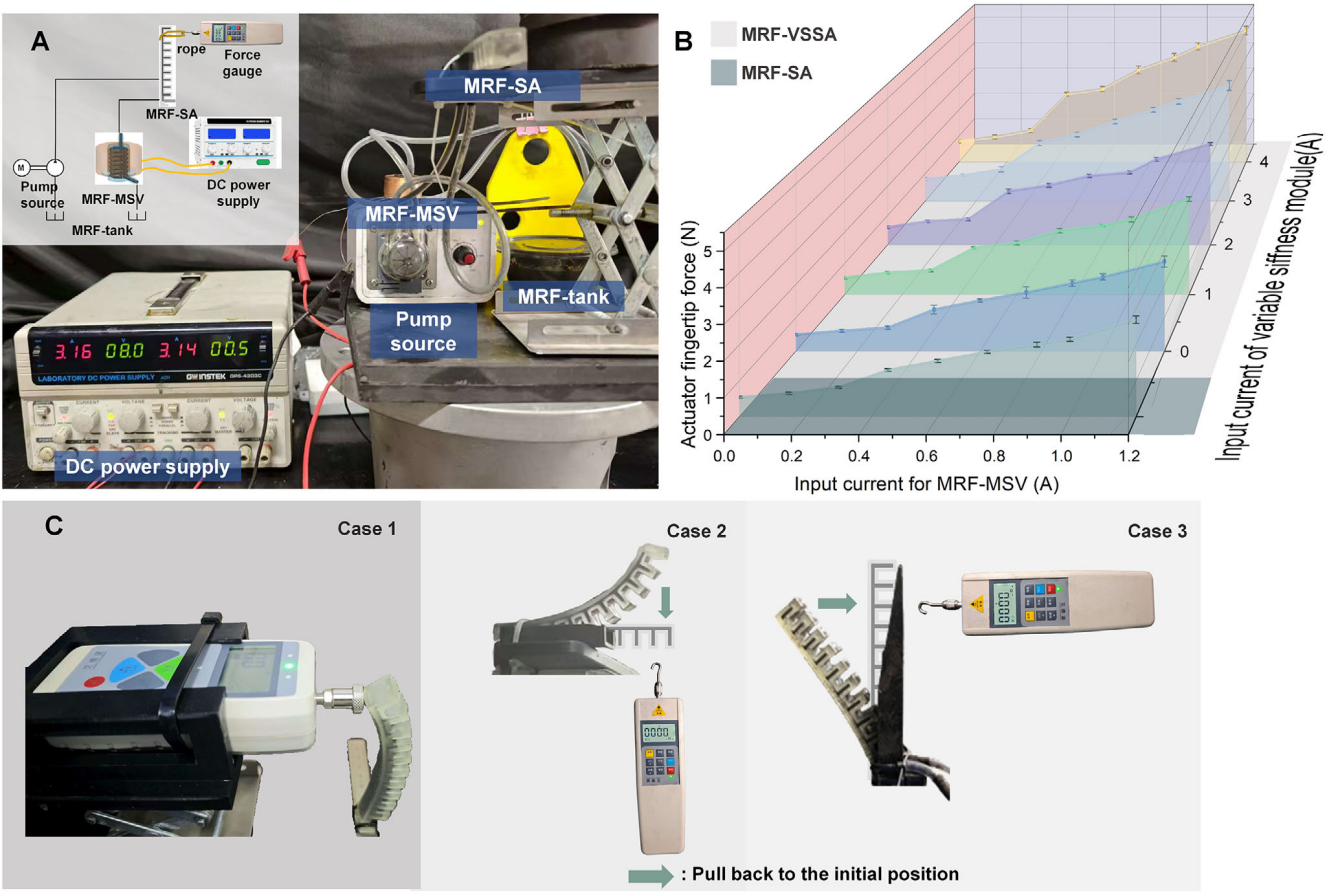
Fingertip force tests for MRF‐SA and MRF‐VSSA based on MRF‐MSV. (A) Hydraulic circuit for fingertip force test. (B) The fingertip force of the MRF‐SA and MRF‐VSSA under different MRF‐MSV input currents and different variable stiffness module input currents. The error bars represent the standard deviation of the mean from five experimental trials. (C) Fingertip force testing methods for MRF‐SA and MRF‐VSSA.

The fingertip force generated by both two actuators under varying resistance pressures is further analyzed. To this end, a magnetorheological fluid‐based hydraulic testing system (Figure 7A) was constructed to measure the fingertip output force of the actuator under MRF‐MSV control.

Figure 7C illustrates three testing approaches for evaluating the fingertip force of a soft actuator. In Case 1, the actuator base is fixed, with the fingertip directly contacting the force gauge. However, due to the lack of fixture support on the actuator's back surface, it tends to bend backward under pressure. This causes part of the driving energy to be converted into deformation rather than effective output force, thereby affecting the accuracy of force measurement results. Cases 2 and 3 employ an alternative strategy: first deforming the actuator under pressure, then using a force gauge and cable to pull it back to its initial position to record the fingertip force. Although custom‐made fixtures enhance testing stability, the significant increase in actuator weight due to magnetorheological fluid injection in Case 2 causes interference with the force signal. After comprehensive consideration, Case 3 was ultimately selected for formal testing.

According to the test protocol of Case 3, the fingertip force of MRF‐SA was compared with that of MRF‐VSSA. Under the same experimental conditions, each MRF‐MSV was tested five times, and the mean and standard deviation were calculated. Figure 7B illustrates the fingertip force of MRF‐SA under varying MRF‐MSV input currents, whilst also comparing the fingertip force of MRF‐VSSA under different MRF‐MSV input currents and varying input currents to the variable stiffness module. Test results indicate that as the input current to the MRF‐MSV increases, both the MRF‐SA and MRF‐VSSA exhibit corresponding enhancements in fingertip force output. When the variable stiffness module remains de‐energized, the fingertip force values of the MRF‐VSSA and MRF‐SA remain largely consistent across different MRF‐MSV input currents, confirming that the introduction of the variable stiffness module itself does not significantly impact the actuator's force output. However, as the input current to the variable stiffness module increased, the fingertip force of the MRF‐VSSA gradually increased. When the current reached 1.2 A, the average maximum fingertips force of the MRF‐SA was 2.69 N, while that of the MRF‐VSSA was 5.05 N, representing an 87.7% increase in maximum output force compared to the MRF‐SA. This result unequivocally validates the active enhancement effect of the variable stiffness module on the actuator's force output, demonstrating its potential application in scenarios requiring grasping force control.

Experimental results demonstrate that adjusting the current effectively controls the driving pressure of the MRF‐MSV, thereby enabling precise regulation of the soft actuator's bending deformation and fingertip output force. However, a pre‐bending deformation was observed in both soft actuators at zero input current, resulting in a non‐zero initial bending angle and fingertip force. This phenomenon originates from a back pressure generated as magnetorheological fluid flows through the MRF‐MSV and test pipeline, as illustrated in Figure S9. This back pressure establishes a static pressure in the actuator chamber, thereby inducing an initial pre‐bending, which constitutes an inherent dead zone within the system's operational range.

In summary, by activating the variable stiffness module, the MRF‐VSSA system increases the force at the fingertip from 2.69 to 5.05 N, representing an 87.7% increase, while sacrificing only a small amount of bending deformation capacity. This demonstrates that the damping‐channel‐based MRF variable stiffness mechanism can significantly enhance force output performance while essentially maintaining the actuator's compliance range, providing an effective approach for active force control in soft grippers during grasping tasks. The mechanism behind this variation in stiffness is that the magnetic field produced by the variable‐stiffness control coil enhances the viscosity of the MRF within the damping channel. Consequently, it restricts the redistribution of the fluid inside the actuator and effectively elevates the actuator's bending modulus during the grasping process. To preliminarily evaluate the controllability of the MRF‐VSSA, PID control simulations were conducted based on the experimentally calibrated current‐bend angle and current‐tip force relationships. The simulation results are detailed in Note S10.

### 2.6 Magnetically Driven Smart Fluid Manipulator

**FIGURE 8:**
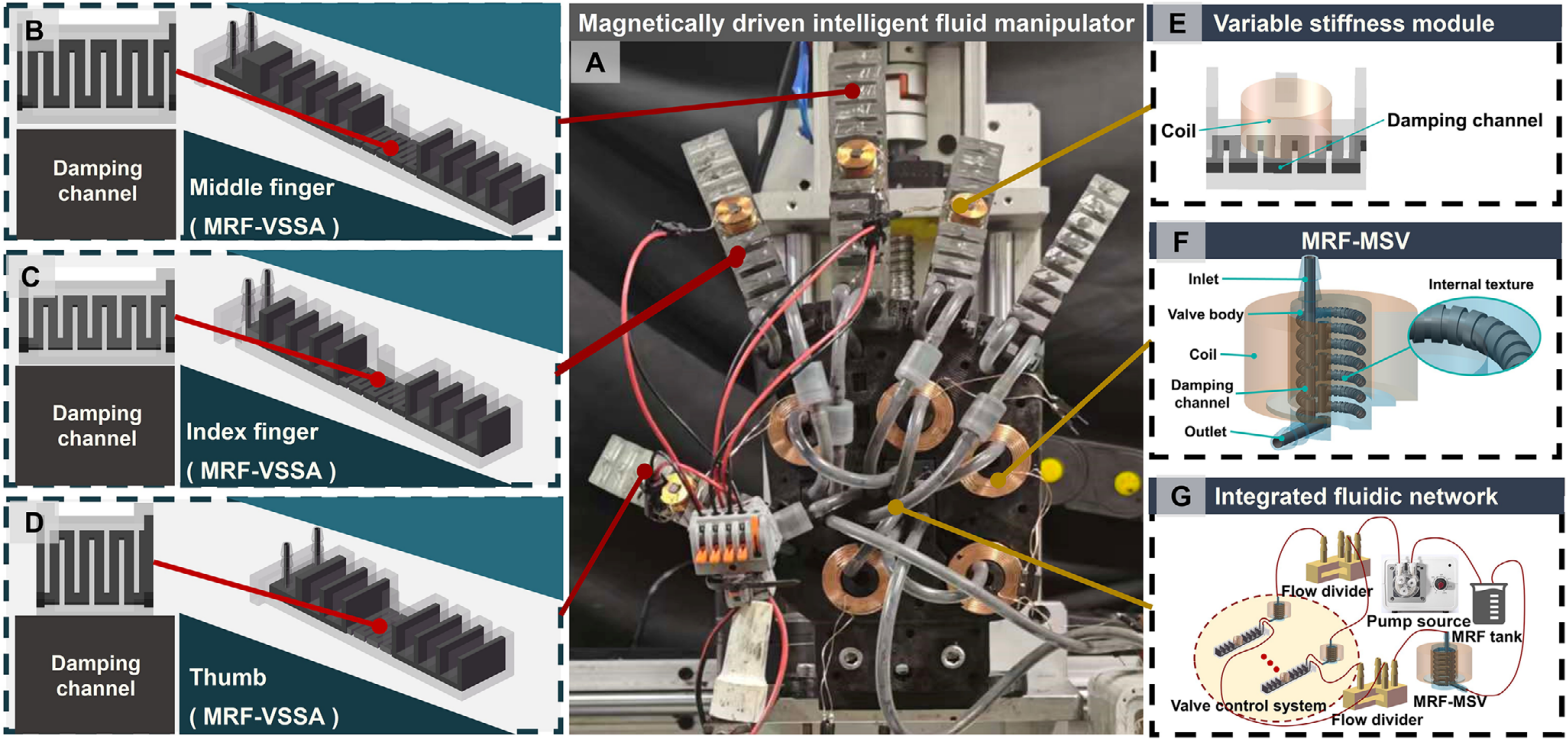
Composition diagram of magnetically driven smart fluid manipulator. (A) Schematic diagram of the magnetically driven smart fluid manipulator prototype. (B–D) MRF‐VSSA configurations for the middle finger, index finger, and thumb respectively, illustrating the damping channel structures within each actuator's variable stiffness module. (E) Core components of each variable stiffness module, comprising coil and damping channel. (F) Five MRF‐MSVs for independent pressure regulation of each finger, with cross‐sectional views illustrating their excitation coils and valve body structures. (G) Integrated channel within the palm for connecting soft actuators, peristaltic pump, MRF‐MSVs, and the MRF tank.

Building upon the aforementioned research into the pressure‐resistance characteristics of MRF‐MSV, as well as the bending deformation and the fingertip mechanical behavior of MRF‐SA and MRF‐VSSA, a magnetically controlled smart fluid flexible manipulator utilizing MRF as the driving medium is proposed. The design of the manipulator draws inspiration from the physiological structural features of the human hand. In human grasping, the five fingers exhibit distinct biomechanical roles. The thumb, serving as the only opposable digit, forms an antagonistic relationship with the other four fingers to establish a force closure. The index finger is responsible for spatial positioning and manipulation guidance, while the middle finger plays a central role in load transfer during forceful grips. The ring finger provides basic stability for grasping through strong adduction, and the little finger, through its coordinated movement with the palm, ensures a secure lock by tightening the lateral edge of the palm against the object [60, 61]. The resulting design strategy is embodied in the key structural features shown in Figure 8A–D.

The thumb, index finger, middle finger, and ring finger utilize an MRF‐VSSA structure to enhance gripping force during grasping tasks performed by the magnetically driven smart fluid manipulator. The little finger employs an MRF‐SA structure, leveraging its superior deformation capability to achieve effective envelopment and locking of target objects during the grasping process. To enhance MRF‐SA performance, the number of chambers has been increased by adding two extra rectangular chambers. Each MRF‐VSSA variable stiffness module comprises a stiffness control coil and a damping channel, with its structure illustrated in Figure 8E. During operation, the magnetorheological fluid within the module undergoes viscosity changes in response to the magnetic field generated by the coil, thereby enabling active stiffness regulation. Simulation results in Figure 6A,C demonstrate that the resistance pressure generated by this module effectively counteracts the maximum driving pressure of the soft actuator, achieving reliable control over system stiffness. Each soft actuator is independently controlled by its corresponding MRF‐MSV, with the relevant structure shown in Figure 8F. The manipulator features a fully integrated fluidic network (Figure 8G) that connects all soft actuators, peristaltic pump sources, MRF‐MSVs, and the MRF tank. This fluidic system is compactly integrated within the palm structure, maximizing overall system integration.

**FIGURE 9:**
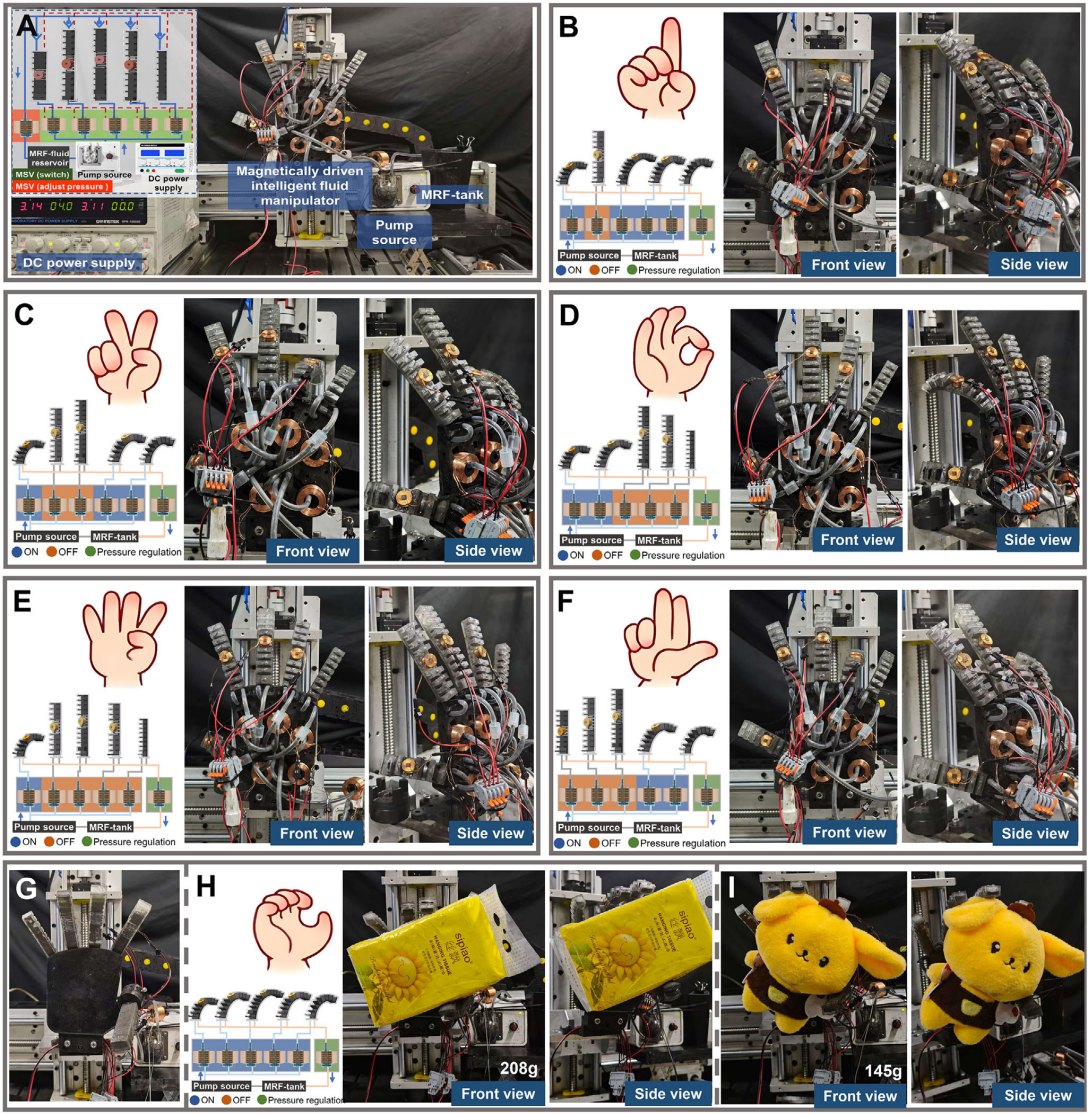
Demonstration of gesture movements and grasping capabilities for the magnetically driven smart fluid manipulator. (A) Schematic illustration of the hydraulic drive principle and the initial state of the manipulator system. (B–F) Experiment on single‐finger and multi‐finger coordinated gesture movements. (G) The manipulator's thumb is adjusted to an opposable position. (H, I) Grasping tests of the manipulator on soft objects.

In the gesture demonstration experiment conducted for the magnetically driven smart fluid manipulator, a hydraulic drive system based on MRF‐15D was first constructed. The overall system architecture is shown in Figure 9A. The workflow begins with a peristaltic pump drawing MRF from the tank and delivering it to five independent MRF‐MSV branches, which in turn controlling the movements of five fingers. Subsequently, the MRF flows through the soft actuators corresponding to each finger and their associated check valves, ultimately converging into the main circuit MRF‐MSV before returning to the tank, thus forming a complete closed‐loop hydraulic circuit.

Within this hydraulic system, the main circuit and branch circuit MRF‐MSVs are respectively responsible for continuous pressure regulation and on‐off control. By adjusting the input current, the main circuit MRF‐MSV allows for continuous control of finger deformation through regulated resistance pressure, whereas the branch circuit MRF‐MSVs function as on‐off valves that fully suppresses MRF flow by applying excitation current exceeding the main circuit's threshold, thereby selectively activating or stopping designated finger movements. The on‐off mechanism is based on the pressure‐resistance characteristic of the MRF‐MSV. By applying high or low input current to the MRF‐MSV, the apparent viscosity of the internal MRF can be actively regulated, allowing for discrete switching of the flow path between “open” and “closed” states and thus achieving independent, discrete control over multi‐finger actions.

Based on the system configuration of the magnetically driven smart fluid manipulator shown in Figure 9A, this study systematically conducted a series of experiments on gesture generation and grasping operations. During the gesture generation phase, precise control over the activation states of branch circuit MRF‐MSVs and pressure regulation of the main circuit MRF‐MSV successfully enabled independent single‐finger movement and coordinated multi‐finger gestures (Figure 9B–F). Each gesture was recorded from both frontal and lateral perspectives to capture the spatial deformation characteristics and action trajectories of the actuator. (Movie S3 shows the manipulator's gesture movements)

To further evaluate the performance of this manipulator in practical operational tasks, this study designed a grasping experiment targeting flexible objects. First, the thumb was adjusted to a biomechanically optimized opposable position, forming a hand configuration capable of enveloping grasping, as shown in Figure 9G. In this grasping mode, the branch circuit MRF‐MSVs corresponding to all fingers remained continuously activated, ensuring a stable flow of MRF between actuators. The main circuit MRF‐MSV dynamically adjusted system pressure in real‐time based on the dimensions and stiffness characteristics of the target object. This controlled the actuators to generate bending deformations, enabling adaptive enveloping and stable grasping of the object. (Movie S4 shows the manipulator grasping objects)

As shown in Figure 9H,I, the manipulator successfully grasped both the tissue box and the plush toy. Owing to its compliant nature, the manipulator passively conforms to the object's surface, which intrinsically adjusts contact force distribution during grasping, ensuring sufficient holding force while preventing damage to fragile items. This series of grasping experiments fully demonstrates the manipulator's excellent shape adaptability and operational compliance when handling objects of varying sizes, materials, and structural characteristics.

Experimental results demonstrate that the magnetically driven smart fluid manipulator developed herein not only accurately and reliably reproduces multiple pre‐programmed gestures, possessing rich gestural expressiveness, but also exhibits superior environmental adaptability and operational capability in practical object‐grasping tasks. As presented in Table 2, the developed magnetically‐controlled smart fluid manipulator incorporates a magnetically‐controlled variable stiffness module and MRF‐MSVs to achieve a working pressure of 350 kPa, a bending angle of 171.2 °, a fingertip force of 5.05 N, and a grasping capacity of 208 g. It either outperforms or matches various mainstream soft actuators listed in the table in aspects of drive capability, deformation capability, grasping capability, and manufacturing process, rendering it especially suitable for flexible manipulation tasks that demand a wide range of bending and moderate grasping force. This research validates the application feasibility and broad prospects of magnetorheological fluid‐based hydraulic technology within the field of flexible robotics, particularly in human‐machine interaction, gestural communication, and grasping operations for service robots.

**TABLE 2:** Comparison of the unique designs and performance characteristics of different manipulators.

Reference	Operating principle	Working medium	Unique design	Maximum pressure	Bending angle / Deformation	Fingertip force / Grip weight
This study	High‐pressure fluid‐driven multi‐chamber elastomer/ Magnetron MRF	MRF	Magnetically driven variable stiffness module	350 kPa	171.2 °/ —	5.05 N/ 208 g
[15]	High‐pressure fluid‐driven multi‐chamber elastomer	Water	—	3 MPa	‐/113.5 mm	— /2 kg
[17]	High‐pressure fluid‐driven multi‐chamber elastomer	Air and water	—	448 kPa	360 ° / —	0.6 N / —
[49].	High‐pressure fluid‐driven elastomer/ Magnetron MRF curing adaptive morphology	Air and MRF	Magnetically driven variable stiffness module	160 kPa	— / —0	5.3 N/‐
[50]	Magnetron MRF curing adaptive morphology	MRF	MR fluid gripper	—	—/ —	50 N/‐
[62]	Combination of tendon‐driven and high‐pressure fluid‐driven elastomers	Air	Pumpless hybrid drive system combining pre‐pressurized air and rope drive	60 kPa	100 °/ —	384 g/‐
[63]	Vacuum‐driven	Air	Retractable suction cup	−50 kPa	— / 72 mm	26.3 N / 10 kg
[14]	Photothermal‐driven shape‐memory materials	—	Light‐controlled local deformation	—	70 °/ —	‐/5 times its self‐weight
[64]	High‐pressure fluid‐driven multi‐chamber elastomer/ Temperature‐controlled alloy	Air	Thermochromic Variable Stiffness Module	20 kPa	— / —	2.7N/780 g

## 3 Discussion

### 3.1 Advantages and Operating Mechanism of MRF Valve Control Systems

This study successfully designed and validated a smart fluid hydraulic system based on magnetorheological fluid (MRF) valve control. Unlike traditional hydraulic valves that regulate pressure by altering flow channel cross‐sectional area (e.g., spool valves, needle valves), the MRF‐MSV in this system achieves pressure resistance by precisely controlling MRF viscosity through a magnetic field. This “valve‐controlled viscosity” mechanism fundamentally simplifies hydraulic system architecture by eliminating the need for intricate mechanical moving parts and replacing them with electro‐magneto‐rheological control. Such a paradigm shift represents not only a methodological innovation but also opens possibilities for achieving integrated structural and functional design in soft robotics. As analogized in the introduction with the phenomenon of vascular embolism in the human body, the MRF‐MSV can smartly interrupt pressure transmission by creating a magnetically controllable “fluid embolism” within the damping channel, representing a highly biomimetic control strategy.

### 3.2 Synergistic Enhancement Effect of Damping Channel Micro‐Texture Structures in MRF‐MSV

Simulation and experimental results demonstrate that introducing rectangular ring micro‐texture structures within the damping channel significantly enhances the pressure resistance capability of the MRF‐MSV. We propose the following mechanism to explain this phenomenon.

#### 3.2.1 Flow Field Disturbance Effect

Micro‐textures disrupt the low‐viscosity boundary layer formed near the channel wall. In untextured channels, this boundary layer provides a pathway for fluid to “slip” under high pressure. Within micro‐texture grooves, however, a high‐viscosity, near‐stagnant MRF lock‐up zone forms, significantly increasing the overall flow resistance of the MRF.

#### 3.2.2 Magnetic Field Concentration Effect

The edges of rectangular micro‐textures may induce changes in the local magnetic field distribution, enhancing magnetic field gradients. This leads to the formation of more stable structures within magnetic particle chains in these regions, thereby increasing the apparent yield stress of the MRF.

#### 3.2.3 Solid‐Liquid Hybrid Behavior

The micro‐texture design transforms the MRF flowing through the channel into a hybrid state, with high‐viscosity, quasi‐solid regions inside the micro‐texture grooves and low‐viscosity, quasi‐liquid regions between adjacent grooves at the microscopic scale, as shown in Table S2. This hybrid behavior is key to achieving high pressure resistance. However, when optimizing micro‐texture spacing and width, a trade‐off exists between manufacturing feasibility and compressive strength.

### 3.3 Integration of Valve Control Systems With Soft Actuators

#### 3.3.1 Simplified System Architecture

Traditional hydraulic or pneumatic systems for soft robotics require separate pumps, valves, controllers, and complex pipeline. This study directly integrates critical pressure regulation with drive functions, enabling simultaneous control of actuator action and stiffness through a single electrical signal, thereby significantly simplifying the system.

#### 3.3.2 Implementation of the Variable Stiffness Mechanism

Experimental results of the MRF‐VSSA validate that valve‐controlled resistance can be directly converted into actuator stiffness. When current is applied, the MRF viscosity within the damping channel of the variable stiffness module increases. This not only restricts MRF flow within the actuator chamber but also fundamentally alters the bending modulus of the entire actuator. It transitions from a soft, easily deformable state to a rigid state capable of sustaining bending deformation and generating greater fingertip force. This explains why MRF‐VSSA exhibits comparable deformation to the MRF‐SA, yet its fingertip force output enhances when the variable stiffness module is activated.

### 3.4 System‐level Application Performance

The final magnetically driven smart fluid manipulator demonstrates the system's potential for complex grasping tasks. By assigning MRF‐VSSA and MRF‐SA structures to different fingers, we mimic the functional division of the human hand. MRF‐VSSA provides robust gripping force and stability, while MRF‐SA leverages its large deformation capability for initial enveloping and locking. This function‐based configuration design demonstrates the excellent reconfigurability and task adaptability of this smart fluidic hydraulic system. The manipulator achieves damage‐free grasping, primarily because the system can precisely regulate grasping force through current adjustment, avoiding damage to fragile objects caused by traditional rigid grippers.

## 4 Conclusions

This study successfully developed a smart fluid‐hydraulic system controlled by a magnetorheological fluid (MRF) and validated its functionality on a soft manipulator. The main achievements and innovations are summarized as follows:

1. By incorporating a rectangular ring micro‐texture into the damping channel of the MRF‐MSV and utilizing the fluid embolism effect based on flow field patterns, continuous proportional flow resistance regulation was achieved without the need for a mechanical spool valve. Under limited magnetic field conditions, the valve body's pressure resistance was increased by 81.9% (from 655 to 1191.4 kPa), achieving a wide pressure regulation range of 60–1191.4 kPa.
2. By integrating a damping channel into the MRF‐SA, a soft actuator with variable stiffness was achieved, with a maximum bending angle of 171.2°. The fingertip force of the MRF‐VSSA increased by 87.7% compared to the MRF‐SA (from 2.69 to 5.05 N), while maintaining similar deformation capabilities.
3. A magnetically controlled smart fluid manipulator was constructed by integrating the MRF‐MSV with soft actuators. This manipulator is capable of performing various complex gestures and stably grasping objects of different shapes, sizes, and materials (such as tissue boxes and plush toys), with a grasping weight of up to 208 g without causing damage. It demonstrates excellent shape adaptability and operational compliance.

Compared with existing mainstream soft actuators and smart fluidic microvalves, this system provides a wider pressure regulation range, larger bending angles, and better force output capabilities. It maintains a compact structure and employs advanced LCD 3D printing manufacturing techniques. However, the proposed magnetically driven smart fluid manipulator has not been integrated with flexible sensors. Although preliminary tests have been carried out on the actuator's bending deformation and fingertip force, the system does not have a closed‐loop control architecture, and intelligent algorithms have not been introduced to achieve real‐time optimization and adaptive control of the grasping process. Future work will concentrate on integrating flexible sensors to establish a closed‐loop control system, optimizing the peristaltic pump and tubing design to enhance the response speed, and developing adaptive control algorithms suitable for dynamic grasping. Nevertheless, this work has laid an important foundation for the further development of the embedded smart drive system of the soft robot.

## 5 Materials and Methods

### 5.1 Design, Materials, and Manufacturing of Soft Manipulator Systems

The soft manipulator employs a peristaltic pump 104BLW (LEFOO Peristaltic Pump, flow rate 4–97 mL/min, Zhejiang LIFU Holding Group Co., Ltd., China) as its pressure pump source. Soft actuators serve as the deformation mechanism of the soft manipulator, while an MRF‐MSV functions as the hydraulic component regulating the driving pressure of the finger‐shaped soft actuators, with MRF‐15D (magnetorheological fluid, Zhixing Technology Nantong Co., Ltd., China) serving as the pressure fluid. The sedimentation stability test of MRF‐15D is shown in Figure S10.

The MRF‐MSV consists of an excitation coil (hollow coil, Beijing Zhongci Wisdom Sensing Technology Co., Ltd., China) and a valve body fabricated via photopolymerization 3D printing using Hard‐Tough Resin (photosensitive resin, Shenzhen Guanghua Weiye Co., Ltd., China).

Soft actuators, including MRF‐SA and MRF‐VSSA. The soft actuators are manufactured via photopolymerization 3D printing using F39T (photosensitive resin, Dongguan Shenshuo Technology Co., Ltd., China). The MRF‐VSSA eliminates a rectangular cavity in the middle section of the traditional finger‐shaped soft actuator. This modification integrates the damping channel and combines it with a stiffness control coil (hollow coil, Beijing Zhongci Wisdom Sensing Technology Co., Ltd., China) to form a variable stiffness module, as shown in Figure S4. Dumbbell specimens were printed using F39T material (Ke Jian Instrument, KJ‐1065F) to test the mechanical properties of the F39T material. Testing complied with ISO 37:2005 requirements, yielding stress–strain relationship data. The Yeoh‐3rd model was established to determine the drive pressure and deformation characteristics of the soft actuator. The theoretical bending deformation model for the soft actuator is provided in the supporting documents.

### 5.2 Magnetorheological Fluid Rheological Properties Test

Magnetorheological fluid exhibits rheological effects under an applied magnetic field, transforming from a liquid to a semi‐solid state with corresponding changes in its properties. Apparent viscosity and shear stress serve as indicators characterizing the properties of magnetorheological fluid. Using a rotational rheometer, the rheological properties of MRF‐15D were investigated under different magnetic field strengths. The testing protocol and results concerning the rheological characteristics of MRF‐15D are detailed in the supporting documents.

### 5.3 Establishment of a Bingham Simulation Model Based on MRF‐15D

Using the Bingham model based on the least squares method, the shear stress of MRF‐15D at different shear rates under varying magnetic field strengths is described. The pressure drop equation for the MRF‐MSV is derived based on the flow operating mode of the MRF. Additionally, the yield stress of MRF‐15D under different magnetic field strengths is characterized through polynomial fitting. A User‐Defined Function (UDF) for the Bingham rheological model of MRF‐15D was established within the Fluent simulation software. The resistance characteristics of MRF‐15D under different pressure conditions within the MRF‐MSV system were investigated. Based on the drive pressure requirements for soft robots, an appropriate excitation magnetic field environment was selected, and the excitation coil was custom‐fabricated accordingly. See the supporting documents for details.

## Author Contributions

L.H. contributed to conceptualization, data curation, software, and writing – original draft. H.J. contributed to conceptualization, methodology, investigation, and writing – review and editing. S.N. contributed to conceptualization, investigation, and writing – review and editing. R.H. contributed to methodology and investigation. F.Y. and Z.M. contributed to supervision.

## Funding

This work was supported by the National Natural Science Foundation of China (Grant Nos. 52475043 and 52475045).

## Conflicts of Interest

The authors declare no conflicts of interest.

## Supporting information




**Supporting File 1**: advs76435‐sup‐0001‐SuppMat.docx.

**Supporting File 2**: advs76435‐sup‐0002‐MovieS1.mp4.

**Supporting File 3**: advs76435‐sup‐0003‐MovieS2.mp4

**Supporting File 4**: advs76435‐sup‐0004‐MovieS3.mp4.

**Supporting File 5**: advs76435‐sup‐0005‐MovieS4.mp4.

## Data Availability

The data that support the findings of this study are available from the corresponding author upon reasonable request.
