# Supplementary material for: A Smart Fluid‐Hydraulic System Based on MRF Valve Control and Its Application in Flexible Grasping
Source: Adv Sci (Weinh). 2026 Jul 3:e76435. Online ahead of print. doi: 10.1002/advs.76435 (PMC13334593; doi:10.1002/advs.76435)
Supplement: Supplementary file 1 — Supporting File 1: advs76435‐sup‐0001‐SuppMat.docx. [file ADVS-9999-e76435-s004.docx]

Supporting Information

**A smart fluid-hydraulic system based on MRF valve control and its application in flexible grasping**

*Linfeng Huo, Hui Ji*, Ruidong Hong, Songlin Nie, Fanglong Yin, Zhonghai Ma*

*Corresponding author. Email: [jihui@bjut.edu.cn](mailto:jihui@bjut.edu.cn)

**This PDF file includes:**

Supplementary Text

**Figs. S1** to **S11**

**Tables S1** to **S3**

**Movies S1** to **S4**

Supplementary Text

**Supplementary Note S1: MRF-15D rheological properties test**

1.1. Testing equipment and methods

This study selected MRF-15D as the pressure fluid, it is necessary to determine the viscosity changes in the magnetorheological fluid under different magnetic field excitation. The rheological properties of MRF-15D were tested using an Anton Paar MCR-301 rotational rheometer (Anton Paar Physica MCR301, Anton Paar GmbH, Austria). The magnetorheological module featured a parallel circular plate geometry comprising a fixed bottom plate and a rotating top plate. Grooves were engraved on the surface to minimize errors caused by wall slip. The gap between the rotor plate and the base plate was set at 1 mm. as shown in **Figure S1A**. The current control module modulated the excitation coil current to generate magnetic fields of varying intensities, which were transmitted vertically through the test gap via the upper magnetic guide frame. Additionally, the Anton Paar MCR-301 equipped with inlet and outlet ports maintained constant temperature in the test gap through an internal water-cooling circulation system. Magnetic field strength is set from zero to 450 mT in 50 mT increments (the upper limit is imposed because testing the rheological properties of MRF-15D at magnetic fields exceeding 450 mT would require torque exceeding the instrument's rated capacity). Key tests include the relationship between magnetic field strength and viscosity, the relationship between shear stress and shear rate, and the relationship between magnetic field strength and yield stress for MRF-15D under varying magnetic field strengths.

1.2. Apparent viscosity

Using the magnetorheological module under varying magnetic field strengths, the apparent viscosity of MRF-15D magnetorheological fluid was obtained by increasing the shear rate logarithmically within the range of 0.001 s⁻¹ to 1000 s⁻¹. As shown in **Figure S1B**, the apparent viscosity of the magnetorheological fluid exhibits a sharp decline within a narrow shear rate range. Even in the absence of an external magnetic field, the MRF exhibits significant non-Newtonian behavior, a phenomenon that is particularly pronounced at lower shear rates. Furthermore, as magnetic field strength increases, the non-Newtonian characteristics of the MRF progressively intensify.

To investigate the properties of MRF-15D magnetorheological fluid under varying magnetic field strengths, the apparent viscosity of the fluid at a shear rate of 0.1 s⁻¹ is shown in **Figure S1C**. The apparent viscosity exhibits a nonlinear trend with magnetic field strength, marked by a distinct inflection point 150 mT. Beyond 150 mT, further increases in magnetic field strength cause a rapid rise in the MRF's apparent viscosity, which refers to a fluid's internal friction coefficient and characterizes the intensity of internal friction within a liquid. In MRF, the apparent viscosity arises from the internal friction between magnetic particles, within the carrier fluid, and at their interfaces.

At magnetic field strengths below 150 mT, magnetic particles in MRF form numerous short magnetic chains under the influence of the magnetic field. The primary internal friction arises from interactions between carrier fluid molecules and these short single chains. When the magnetic field strength exceeds 150 mT, magnetic particles form extensive, long, and complete multi-chain structures under the magnetic field. The predominant internal friction then stems from interactions between magnetic particles within these chains, which are significantly stronger than those between carrier fluid molecules and short single chains. As a result, the apparent viscosity of the MRF rises rapidly with increasing magnetic field strength.

1.3. Shear stress

Using the magnetorheological module, the shear stress of MRF-15D was obtained at different magnetic field strengths as the logarithm of the shear rate increased within the range of 0.001 s⁻¹ to 1000 s⁻¹. As shown in **Figure S1D**, the shear stress grows markedly with enhanced magnetic field strength. Under a fixed magnetic field, it increases with shear rate in a progressively diminishing manner and finally plateaus. From a microscopic perspective, increased magnetic field strength enhances the stability of magnetic particle chains. As shear is applied, particle chains gradually tilt under shear force, increasing the distance between particles within the same chain. Once this distance exceeds the effective range for magnetic attraction, the chains fracture. Macro-scopically, this manifests as the MRF reaching yield stress and initiating flow. As the shear rate continues to increase, particle chains undergo repeated fracture and reorganized. When the number of fractured and reorganized chains reaches a dynamic equilibrium, the shear stress stabilizes with increasing shear rate.

As shown in **Figure S2A**, under the influence of an external magnetic field, magnetic particles align in a straight chain configuration along the magnetic field direction. As the magnetic field strength increases, the dipole moments between magnetic particles gradually grow, causing adjacent particles to attract each other more strongly. This results in the particle chains gradually thickening, exhibiting a progression from single-row chains to double-row chains and ultimately to multi-row chains. As the particle chains thicken, each chain contains more magnetic particles. This effectively fills the voids created by increased particle spacing during shear, keeping the distance between adjacent magnetic particles within the range where magnetic attraction persists. Consequently, the probability of chain breakage decreases, enhancing the shear resistance of the MRF.

1.4. Flow Patterns in the MRF of the MRF-MSV

The operating mode of the MRF in the MRF-MSV is the flow mode. In this mode, driven by pressure, MRF flows axially through a meandering, damped channel, while the magnetic field generated by the excitation coil is perpendicular to the flow direction, as shown in Figure S2C. In this mode, the magnetic field induces the magnetic particles to form a chain-like structure connected to the channel walls, generating a field-dependent yield stress opposite to the flow direction. According to the Bingham plasticity constitutive model (**Equation S1**), the yield stress increases as the applied magnetic field strengthens. To clearly illustrate the fluid trajectory, arrows have been added in **Figure S2B** to indicate the complete flow path of the MRF from the inlet end, through the meandering damped channel, to the outlet end. This flow configuration allows the MRF-MSV to generate controllable pressure resistance without any moving mechanical parts, distinguishing it from conventional valves.

1.5. Establishment of the MRF-15D constitutive relationship based on Bingham model

The Bingham model is designed for fluids that begin to flow only after the applied shear stress exceeds a certain critical value (yield stress).

The Bingham model evolved from the Newtonian fluid model, with its constitutive equation being:

|  | (S1) |
| --- | --- |

Where $\tau$ represents the shear stress of the magnetorheological fluid, $\tau_{0}(B)$ denotes the shear yield stress under an applied magnetic field, $\eta_{0}$ indicates the zero-field viscosity of the magnetorheological fluid, and signifies the shear rate. The Bingham model assumes that after yielding, the magnetorheological fluid behaves as a Newtonian fluid, with its shear stress increasing linearly with shear rate while maintaining constant viscosity.

For flow patterns in circular tubes, the total pressure drop $\Delta P$ of the MRF-MSV can be decomposed into the sum of the viscous component $\Delta P_{vis}$ and the magnetically controlled component $\Delta P_{MR}(B)$^[1]^.

| $\Delta P=\Delta P_{vis}+\Delta P_{MR}(B)$ | (S2) |
| --- | --- |

The viscous component $\Delta P_{vis}$ arises from friction losses due to the MRF’s own plastic viscosity and is independent of the magnetic field. For a circular damped channel (diameter *D*, length *L*), the viscous pressure drop at a volumetric flow rate *Q* can be approximated as:

| $\Delta P_{vis}=\frac{128\eta_{0}QL}{\pi D^{4}}$ | (S3) |
| --- | --- |

This value corresponds to the pressure measured when the excitation current is zero.

The magneto constrictive component $\Delta P_{MR}(B)$ is generated by the yield stress induced by a magnetic field, and its general expression is:

| $\Delta P_{MR}(B)=c\cdot\frac{\tau_{0}(B)L}{D}$ | (S4) |
| --- | --- |

Here, *c* is an empirical coefficient related to the cross-sectional shape of the flow channel. $\tau_{0}(B)$ increases as the magnetic field strength increases, resulting in a higher total pressure drop.

Using the Bingham model and least squares method, the shear stress of MRF-15D at different shear rates under varying magnetic field intensities was characterized. **Figure S1E** displays the yield stress of MRF-15D under different magnetic field strengths, fitted using a polynomial fit. The User-Defined Function (UDF) for the Bingham rheological model of MRF-15D was edited in the Fluent simulation software, invoking the MHD model to establish an MRF-15D material model based on the Bingham rheology. The study investigated the resistance characteristics of MRF-15D under varying drive pressures within an MRF-MSV. Based on the drive pressure requirements for the soft actuator, an appropriate excitation magnetic field environment was selected, and the excitation coil was custom-fabricated accordingly.

Supplementary Note S2: Procedure for fabricating a magnetically controlled resistance demonstration model

To validate the controllable flow characteristics of magnetorheological fluid within soft actuator systems, this study designed and fabricated a visual demonstration model comprising two flexible chambers connected by a curved flow channel. The model was fabricated using the 3D-printed sacrificial mold method: a positive mold incorporating the internal cavity structure was produced via 3D printing. Subsequently, equal volumes of silicone components A and B were thoroughly mixed before being poured into the mold and left to cure for six hours. Subsequently, the cured silicone flexible model was removed and filled with MRF-15D magnetorheological fluid. Finally, the filling port and seams were sealed and bonded, yielding a complete magnetically controlled impedance demonstration model, as shown in **Figure S3**.

**Supplementary Note S3: Structure design and performance of excitation coils**

Two locations utilize coils in this study: one in the MRF-MSV, where its magnetic field regulates the MRF viscosity in the damping channels to create pressure resistance; while another in the MRF-VSSA's variable stiffness module adjusts the MRF viscosity in integrated channels to enable active stiffness modulation of the soft actuator.

The MRF-MSV is constructed with an excitation coil wrapped around the valve body, as shown in **Figure S4A**. The excitation coil is wound with 0.5 mm diameter enameled copper wire, rated for a maximum current of 3.97 A. To match the valve body dimensions, as shown in **Figure S4B**, the coil's external dimensions are designed with an inner diameter *φ_c_* of 16.00 mm, an outer diameter *Φ_c_* of 31.50 mm, and a height *H_c_* of 17.00 mm. After winding, the excitation coil has 485 turns, with a resistance value of 3.3$\Omega$ at room temperature (25°C). The correspondence between the excitation coil current and the induced magnetic field strength is shown in **Figure S4C**. The magnetic field generated by the excitation coil is concentrated in the area around its inner axis, which aligns precisely with the location of the valve body (**Figure S4D**).

The MRF-VSSA variable stiffness module consists of a damping channel (**Figure S5B**) integrated within a soft actuator combined with a coil (**Figure S5C**), as shown in **Figure S5A.** **Figure S5C** illustrates the coil configuration, with specific dimensions: the inner ring has a side length *d_CVS_* of 4 mm forming a square, the outer diameter *R_CVS_* is approximately 8 mm forming a circle, and the height *H_CVS_* is 8 mm. The wound coil comprises 260 turns with a resistance value of 0.5$\Omega$ at room temperature (25°C). When energized, the magnetic field generated at the end face of the coil (**Figure S5D**) within the variable stiffness module will excite the magnetorheological fluid in the damping channel. The relationship between the coil input current and the corresponding excitation magnetic field is illustrated in **Figure S5E**.

In this study, all excitation coils were operated in intermittent mode, with extremely short energization times and sufficient cooling periods allowed after each test. Under these conditions, the temperature rise caused by Joule heating was very limited. The operating temperature range of the MRF-15D is -40 °C to +150 °C, and the heat deflection temperature of the valve body’s resin material is 80 °C, which is significantly higher than actual operating conditions. Therefore, temperature effects can be temporarily disregarded.

**Supplementary Note S4: Modeling of bending deformation for soft actuators**

4.1. Calibration of a Yeoh-3rd constitutive model for F39T material

The soft actuator was manufactured using photopolymerization 3D printing technology with flexible transparent photopolymer resin F39T as the fabrication material. In finite element simulation analysis, the choice of hyperplastic deformation prediction method significantly impacts the accuracy of analytical results. The Yeoh model offers advantages such as simplicity, fewer parameters, and the ability to derive a reasonable numerical structure based on minimal uniaxial tensile test data. It is defined by the following elastic strain energy function:

| $W=\sum_{i=1}^{N} C_{i0}\left(\bar{I}_{1}-3\right)^{i}+\sum_{k=1}^{N} \frac{1}{D_{k}}(J-1)^{2k}$ | (S2) |
| --- | --- |

where *J* is the ratio of the volume after deformation to the volume before deformation; *I*_1_ is the first shear tensor invariant; *N*, *C_i_*_0_, and *D_k_* are material constants.

Assuming the F39T material is isotropic and incompressible (*J*=1), the Yeoh 3rd-order model adopted in this study expresses the strain energy density function *W* as:

| $W=C_{10}\left(I_{1}-3\right)+C_{20}\left(I_{1}-3\right)^{2}+C_{30}(I_{3}-3)^{3}$ | (S3) |
| --- | --- |

Uniaxial tensile tests on F39T material were conducted in accordance with the Chinese national standard GB/T 528-2009 "Determination of Tensile Stress-Strain Properties of Vulcanized Rubber or Thermoplastic Rubber". For F39T material, Type 4 dumbbell-shaped standard samples were used, as shown in **Figure S6A**. The F39T test sample has a total length of 35.0 mm, an end width of 6.0 mm, a narrowed section length of 12.0 mm, and a narrowed section width of 2.0 mm. The test sample is fabricated using light-curing 3D printing technology.

The tensile procedure for the F39T test sample is shown in **Figure S6B**. Each sample was subjected to five tensile tests. Starting from the initial state, the sample was stretched at a constant speed of 200 mm/min until fracture, while displacement and tensile force data were continuously recorded. The test was carried out using a tensile-compression testing machine (Guangdong Kejian Instruments Co., Ltd., China), featuring a maximum stroke of 500 mm and a maximum load capacity of 500 N. The resulting tensile data were fitted using MatEditor software to determine the Yeoh third-order hyperelastic material constants (as shown in **Table S3**).

4.2. Theoretical calculation of single-chamber deformation characteristics

The constant curvature per segment assumption employs differential concepts to divide the soft actuator into continuous small segments. Within each small length range, each segment forms an arc with a specific curvature. Consequently, the overall bending of the soft actuator can be viewed as the connection of arc segments formed by the bending of each individual chamber. The deformation principal diagram is shown in **Figure S7A**, where the central angle corresponding to the bending deformation of a single chamber is defined as the bending angle, and the total central angle corresponding to the deformation is defined as the total bending angle of the actuator (assuming identical deformation for each chamber under the same input pressure).

|  | (S4) |
| --- | --- |

Here, *k* denotes the number of cavities in the soft actuator.

The schematic diagram of a single-chamber bending is shown in **Figure S7B**. The arc radius *R* can be calculated as follows.

| $R=\frac{d}{2\sin(\theta/2)}$ | (S5) |
| --- | --- |

Here, *d* corresponds to the chord length of the single-cavity arc after deformation.

Assuming F39T material is isotropic and incompressible, its intrinsic structure is established based on stress-strain theory and represented by the strain energy density function *W*.

| $W=W\left(I_{1},I_{2},I_{3}\right)=\sum_{k=1}^{n} C_{k}\left(I_{1}-3\right)^{k}$ | (S6) |
| --- | --- |
| $\left\{\begin{matrix} I_{1}=\lambda_{1}^{2}+\lambda_{2}^{2}+\lambda_{3}^{2} \\ I_{2}=\lambda_{1}^{2}\lambda_{2}^{2}+\lambda_{2}^{2}\lambda_{3}^{2}+\lambda_{1}^{2}\lambda_{3}^{2} \\ I_{3}=\lambda_{1}^{2}\lambda_{2}^{2}\lambda_{3}^{2}\equiv1 \end{matrix}\right.$ | (S7) |

Among these, *I*₁, *I*₂, and *I*₃ are deformation tensor invariants, while *λ*₁, *λ*₂, and *λ*₃ represent the principal strain ratios in the chamber's length, width, and height directions, respectively. Considering the material's incompressibility, it is assumed that the soft actuator does not deform in the width direction, i.e., *λ*₃ = 1 and *I*₃ = 1. Then, we obtain:

| $\lambda_{2}^{2}=\frac{1}{\lambda_{1}^{2}}$ | (S8) |
| --- | --- |
| $I_{1}=I_{2}=\lambda_{1}^{2}+\frac{1}{\lambda_{1}^{2}}+1$ | (S9) |

According to Yeoh's 3rd-order model, selecting a typical binomial parameter form, substituting equations (S7) into the following expression, the strain energy density function model can be expressed as:

| $\begin{matrix} W=C_{10}\left(I_{1}-3\right)+C_{20}\left(I_{1}-3\right)^{2}+C_{30}\left(I_{1}-3\right)^{3} \\ =C_{10}(\lambda_{1}^{2}+\frac{1}{\lambda_{1}^{2}}-2)+C_{20}(\lambda_{1}^{2}+\frac{1}{\lambda_{1}^{2}}-2)^{2}\text{+}C_{30}(\lambda_{1}^{2}+\frac{1}{\lambda_{1}^{2}}-2)^{3} \end{matrix}$ | (S10) |
| --- | --- |

where *C_10_*、*C_20_* and *C_30_* are material constants.

Since each chamber bends to the same angle during actuator bending, the total bending angle of the soft actuator can be calculated from **Equation S2**. This simplifies its bending characteristic analysis to that of a single chamber. The structural parameters of the single chamber along the length direction are shown in **Figure S7C**, while those along the width direction are shown in **Figure S7D**. *l*₁, *h*₃, and *w*₁ denote the wall thicknesses in three directions; *l*₂, *h*₂, and *w*₂ denote the chamber lengths in three directions; *h₁* is the thickness of the chamber base; *l_3_* is the spacing between chambers; *h₄* is the height of the chamber connection section; and *h₅* is the height of the channel within the chamber.

Assuming that the self-weight of the MRF-SA is neglected during deformation and no external forces are applied, the sum of the work done by the system and the energy stored is zero for any infinitesimal virtual displacement. According to the principle of virtual work, the work done by the driving hydraulic pressure *p* is completely converted into stored energy within the actuator after deformation.

| $p\text{d}V_{a}=V_{r}\text{d}W$ | (S11) |
| --- | --- |

where *V*_a_ is the volume of the chamber, and *V*_r_ is the volume of the thermoplastic material after deformation.

Assuming the F39T material is incompressible, its volume remains unchanged before and after deformation, as can be derived:

| $V_{r}=lhw+(l_{1}+l_{3})(h_{1}+h_{4})w-l_{2}h_{2}w_{2}$ | (S12) |
| --- | --- |
| $l=2l_{1}+l_{2}$, | (S13) |
| $h=h_{1}+h_{2}+h_{3}$, | (S14) |
| $w=2w_{1}+w_{2}$ | (S15) |

The volume of the chamber after deformation is:

| $V_{a}=V-V_{r}$ | (S16) |
| --- | --- |

The approximate calculation result is:

| $V_{a}=\frac{(1+\lambda)[lh+l_{3}(h_{1}+h_{4})]w}{2}-V_{r}$ | (S17) |
| --- | --- |

The elongation in the chamber length direction can be expressed as:

| $\lambda\text{=}\frac{\theta}{\sin\theta}$ | (S18) |
| --- | --- |

Substituting **Equations S12** and **S17** into **Equation S11** yields a mathematical model relating the input pressure to the structural parameters.

| $p=\frac{2\sin^{2}\theta[lhw+(l_{1}+l_{3})(h_{1}+h_{4})w-l_{2}h_{2}w_{2}]}{[lh+l_{3}(h_{1}+h_{4})]w(\sin\theta-\theta\cos\theta)}\cdot\frac{\text{d}W}{\text{d}\theta}$ | (S19) |
| --- | --- |

In **Equation S19**, *dW*/*dθ* can be expressed as a function containing only *θ*. Thus, the equation contains only two unknown variables, *p* and *θ*. The required MRF pressure can be determined by establishing the bending angle *θ* of the single chamber.

4.3. Theoretical calculation of multi-chamber deformation characteristics

As shown in **Figure S7E,** establish a rectangular coordinate system with the origin at the starting point of the bending deformation curve, the x-axis aligned with the bending direction of the actuator, and the y-axis aligned with the length direction of the actuator. Assume there are *k* chambers in total, the total bending angle *φ* is the sum of the deformation angles of each individual chamber.

|  | (S20) |
| --- | --- |

Supplementary Note S5: Deformation simulation of flexible grippers and soft trunk actuator

**Figure S8** illustrates the deformation process and chamber structural response of the flexible gripper (**Figure S8A**) and soft trunk actuator (**Figure S8B**) under internal fluid pressure through finite element simulations. Under pressure loading, the flexible gripper's cavity undergoes inflation and directional bending, with simulation results reflecting the deformation mechanism underlying its pinching action. The soft trunk actuator's cavity exhibits axial gradient expansion coupled with overall curling deformation, revealing the deformation patterns enabling its curling motion.

**Supplementary Note S6: Driver backpressure testing for MRF-SA**

It was observed that the MRF-SA exhibited initial bending without any adjustment to the MRF-MSV. This led to the inference that a certain initial back pressure exists within the hydraulic system when the magnetorheological fluid flows through it in the absence of an external magnetic field. Test results (**Figure S9**) indicate this back pressure value is approximately 60 kPa. Further analysis indicates that the back pressure is mainly attributed to the flow resistance in the damping channel of the valve body, while the contribution from pipeline flow is negligible.

**Supplementary Note S7: Simulation of MRF viscosity under pressure in smooth and microtextured damping channels**

Simulation analyses were performed to investigate the viscosity variation of magnetorheological fluid entering smooth and microtextured damping channels under different pressures, as detailed in **Tables S2** and **S3**, respectively.

**Supplementary Note S8:** **Experimental observation of the sedimentation characteristics of MRF-15D**

To evaluate the sedimentation stability of MRF-15D magnetorheological fluid in practical applications, it was injected into a transparent glass test tube (inner diameter 12 mm, liquid column height approximately 55 mm), and allowed to stand at room temperature (25±1°C). The sedimentation was observed and recorded at the initial state (0 h) and after standing for 3 h, 6 h, 9 h, 12 h, and 15 h, as shown in **Figure S10**.

The experimental results show that after 3 h of standing, a very slight clear layer began to appear on the surface of MRF-15D, but the amount of sedimentation was negligible; as the standing time was extended to 9 h, the height of the clear layer increased slowly, but most of the magnetic particles remained in suspension; by 15 h, the height of the clear layer accounted for approximately 19% of the total height of the liquid column, and no hard settling occurred. Gently shaking the test tube allowed the settled particles to completely redisperse, restoring a uniform state.

The experiments demonstrate that MRF-15D exhibits good sedimentation stability within the typical experimental timeframe and is suitable for the soft robotics operation scenarios in this study. For situations requiring longer periods of standing (e.g., several hours or more), stirring is necessary prior to use to redisperse the particles.

**Supplementary Note S9:** **Relationship between MRF-MSV pressure drop and MRF-VSSA fingertip force as a function of input current**

As the input current increased from 0 A to 3.0 A, the pressure drops across the MRF-MSV rose from approximately 0 kPa to 1191.4 kPa. As the input current increased from 0 A to 1.2 A, the fingertip force of the MRF-VSSA increased from approximately 0.8 N to 5.05 N. Both parameters increased monotonically with increasing current and exhibited an approximately linear relationship across the entire current range. These results indicate that by adjusting the input current of the MRF-MSV, continuous control of both system pressure and actuator tip force can be achieved simultaneously. The gray shaded area in the figure (current > 1.2 A) represents the operating range exceeding the flexible actuator’s safe pressure limit (350 kPa); it is recommended to avoid operation in this region in practical applications. The data points represent the average of five independent trials, and the standard deviation has been calculated.

**Supplementary Note S10: Simulation of PID control for the bending angle and fingertip force of the current-controlled MRF-VSSA**

To preliminarily verify the controllability of the MRF-VSSA, we conducted PID control simulations based on the experimentally measured relationships between input current and bending angle, and between input current and fingertip force, as shown in **Figure S12**. Using system identification methods, we established second-order transfer function models for the bending angle channel and the fingertip force channel, respectively. The model structure is as follows:

| $G(s)=\frac{K\omega_{n}^{2}}{s^{2}+2\xi\omega_{n}s+\omega_{n}^{2}}e^{-\tau s}$ | (S21) |
| --- | --- |

Here, *K* is the steady-state gain, $\omega_{n}$ is the natural frequency, $\xi$is the damping ratio, and $\tau$ is the pure delay. The PID controller parameters were tuned. The simulation was performed in the MATLAB/Simulink environment using a fixed-step solver, and the simulation time covered the entire process until the system reached steady state.

(1) Bending Angle Control

The target bending angle was set to 150°. The PID control simulation curve shows that the system rises rapidly from 0°, reaching the target value within approximately 10 seconds. The overshoot is about 10%, the steady-state error is less than 5°, and the steady-state error is controlled within 3%. This indicates that the designed PID controller can accurately and rapidly drive the MRF-VSSA to achieve the desired bending angle.

(2) Fingertip Force Control

The target fingertip force was set to approximately 4 N. The PID simulation curve also demonstrated good tracking performance: the force value rose to the target value within approximately 0.5 s, with an overshoot of about 10% and a steady-state error of less than 0.1 N. This indicates that precise control of the fingertip force can be achieved by adjusting the input current.

Regarding the selection of PID control gains, the PID parameters were tuned and subsequently fine-tuned iteratively in Simulink to meet the performance specifications: overshoot $<10\%$, and steady-state error $<3\text{°}$ (bend) or $<0.1$ N (force). For the bending angle control loop, which has significant inertia, a relatively conservative $K_{p}$ value was selected to avoid overshoot and oscillations. For the fingertip force control loop, which exhibits a faster response due to the MR effect, a higher $K_{p}$value was adopted to accelerate the rise time, while the integral gain was carefully limited to prevent integrator oscillations caused by the MR’s rapid response.

It is worth noting the significant difference in response time between bending angle control (≈10 s) and fingertip force control (≈0.5 s) observed in the PID simulations (**Figure S12**). This disparity originates from the distinct physical mechanisms governing each variable.

The bending angle response is dominated by the fluid-structure interaction during the inflation process of the soft actuator. Given that the peristaltic pump supplies MRF at a restricted flow rate (25-50 mL/min), the inflation of the chamber and the elastic deformation of the F39T material demand a significant amount of time, leading to a large mechanical time constant. In contrast, the fingertip force in the MRF-VSSA is not mainly determined by the injection rate. Instead, as detailed in **Section 2.5**, force amplification is accomplished by activating the variable stiffness module after the actuator has reached its target bending position. Once the stiffness control coil is energized, the viscosity of the MRF increases rapidly, obstructing the damping channel and preventing the high-pressure MRF from flowing back. This eliminates the internal fluid redistribution caused by the adaptive deformation of the actuator when it comes into contact with an object and promptly increases the force exerted by the fingertip. Therefore, the faster force response reflects the inherent rheological dynamics of the MRF, while the slower bending response reflects the overall hydraulic charging dynamics of the soft actuator. This temporal separation supports a two-stage control strategy, in which coarse positioning is initially achieved through main damping control, followed by rapid grip force adjustment via the variable stiffness current.

The simulation results validate the PID controller’s ability to effectively regulate the bending angle and fingertip force of the MRF-VSSA, demonstrating the feasibility of continuously controlling grasping force and deformation through current regulation. The current simulation serves as a theoretical validation under an open-loop model; in the future, flexible sensors will be integrated to construct an actual closed-loop system for further experimental validation.

Supplementary References

1. A. Grunwald, A.G. Olabi, Design of magneto-rheological (MR) valve. *Sensor Actuat A-Phys*. 148(1), 211-23. (2008). https://doi.org/10.1016/j.sna.2008.07.028.

|  |
| --- |
| **FIGURE S1.** Rheological property testing of MRF-15D. (A) Principle of the rotational rheometer and MRF testing apparatus. (B) Relationship between shear rate and apparent viscosity of MRF-15D under different magnetic field strengths. (C) Relationship between magnetic field strength and apparent viscosity of MRF-15D at shear rates of 0.1–1 s⁻¹. (D) Relationship between shear rate and shear stress of MRF-15D under different magnetic field strengths. (E) Relationship between magnetic field strength and yield stress of MRF-15D. |

| 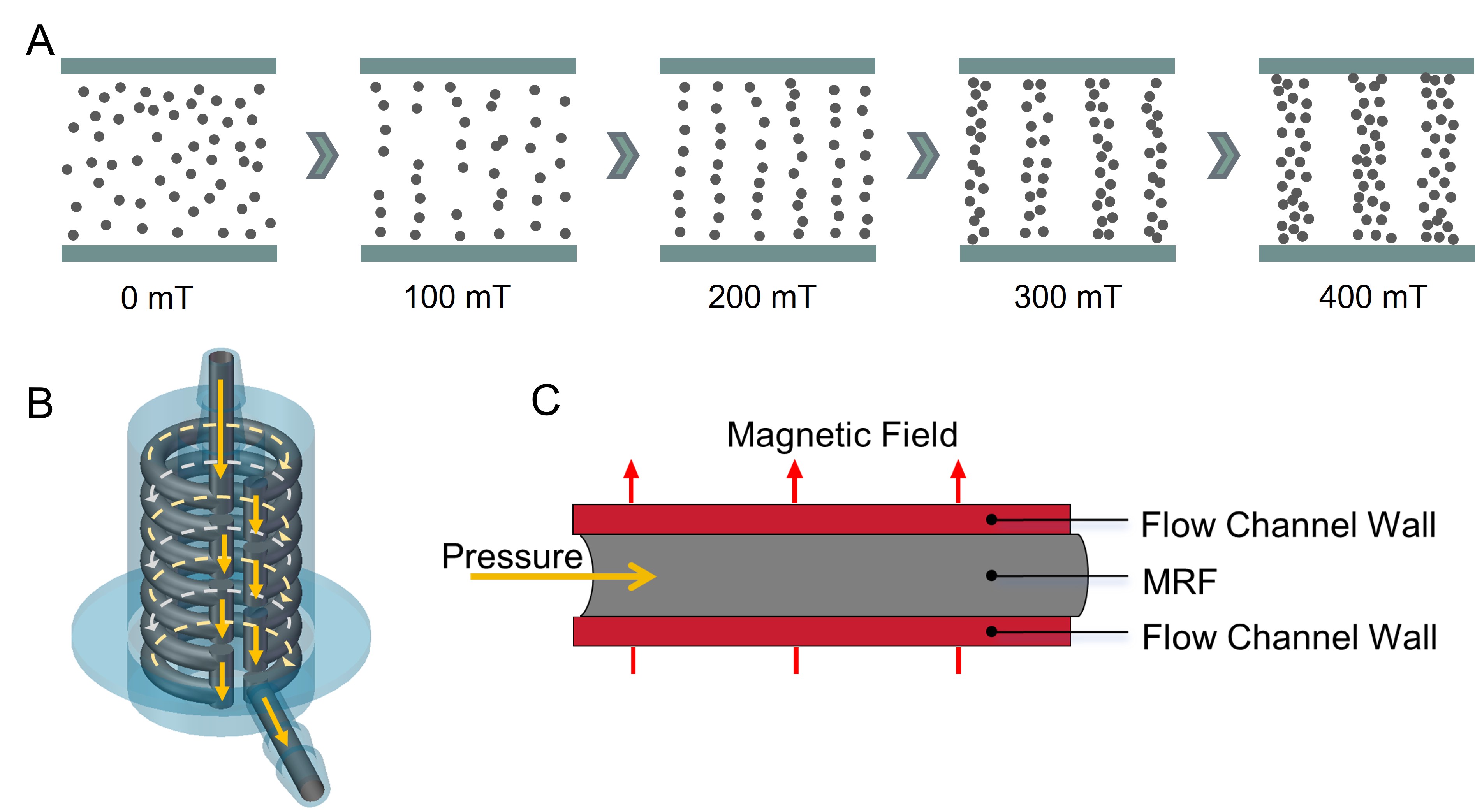 |
| --- |
| **FIGURE S2.** Schematic illustration of the magneto strictive structural evolution and stress distribution of MRF in flow mode. (A) Changes in the chain-like structure of magnetic particles in MRF under different magnetic field strengths (0–400 mT). (B) Flow path of MRF within the MRF-MSV. (C) Flow model of MRF. |

| 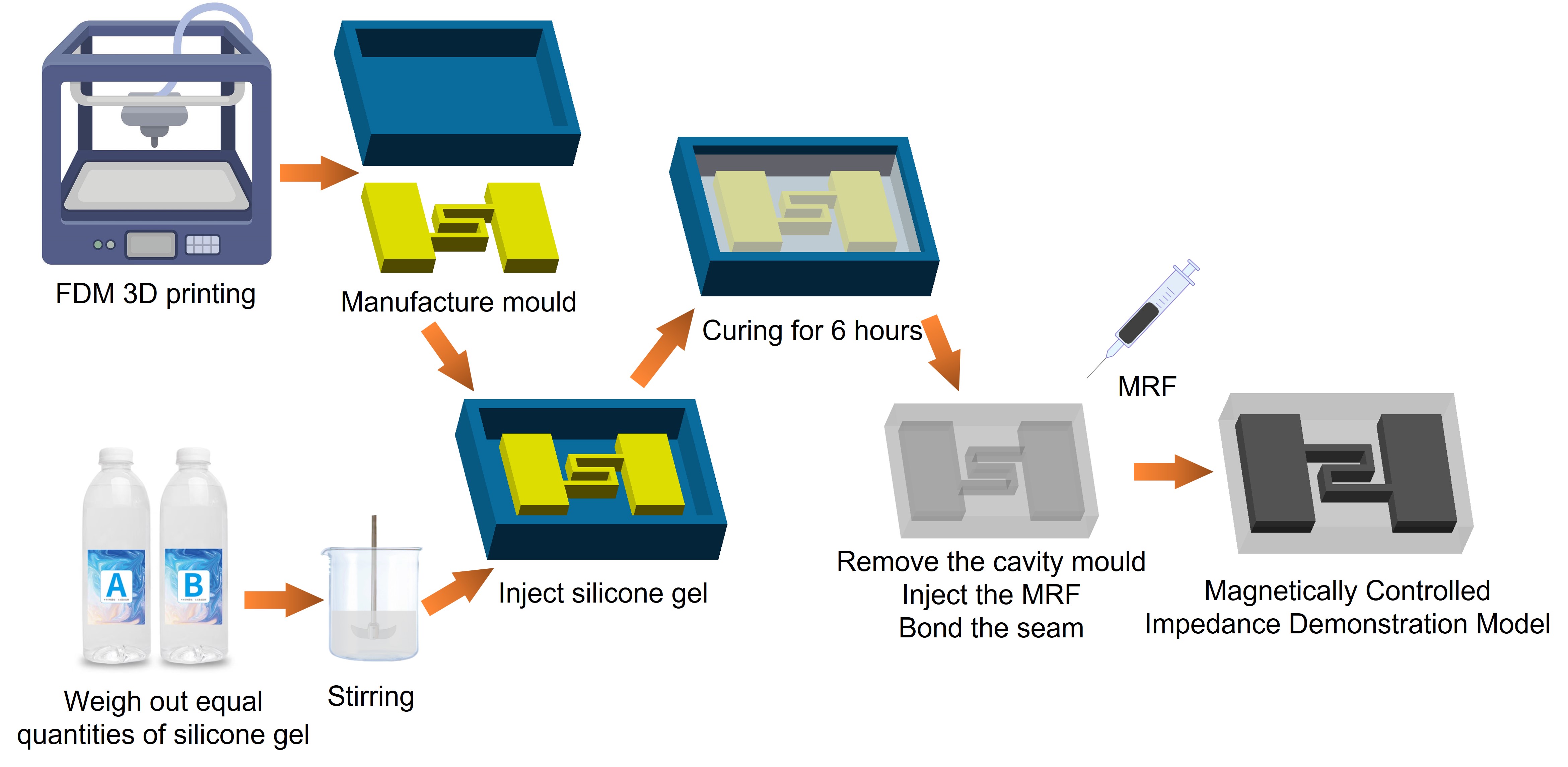 |
| --- |
| **FIGURE S3**. Fabrication process of the magnetically controlled impedance demonstration model. |

| 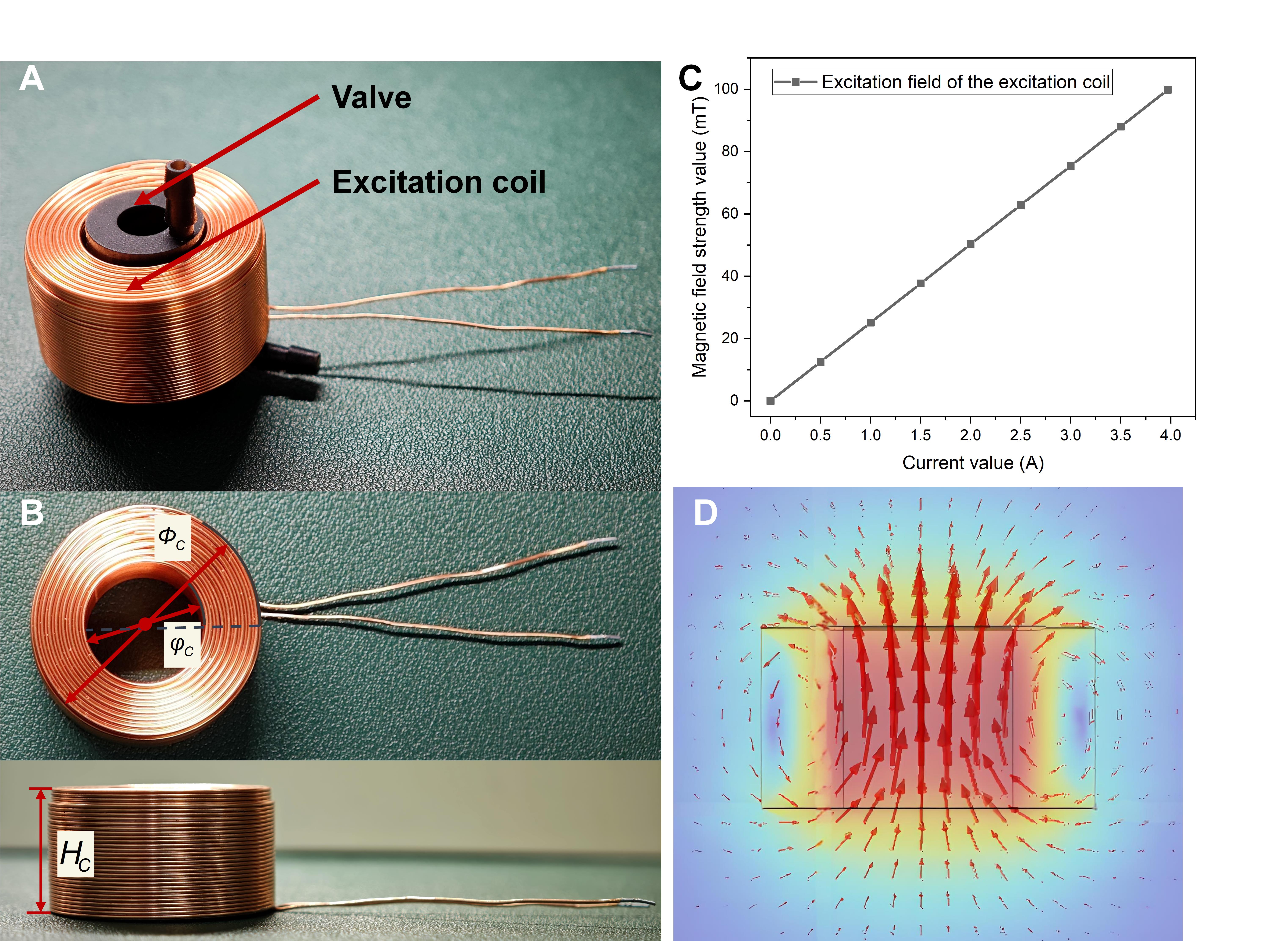 |
| --- |
| **FIGURE S4**. Coil configuration for a magnetorheological fluid-based MRF-MSV. (A) Spatial relationship between valve body and coil within the MRF-MSV. (B) Schematic of key structural parameters for the coil. (C) Relationship between coil input current and response magnetic field. (D) Distribution of the simulated magnetic field generated by the coil. |

| 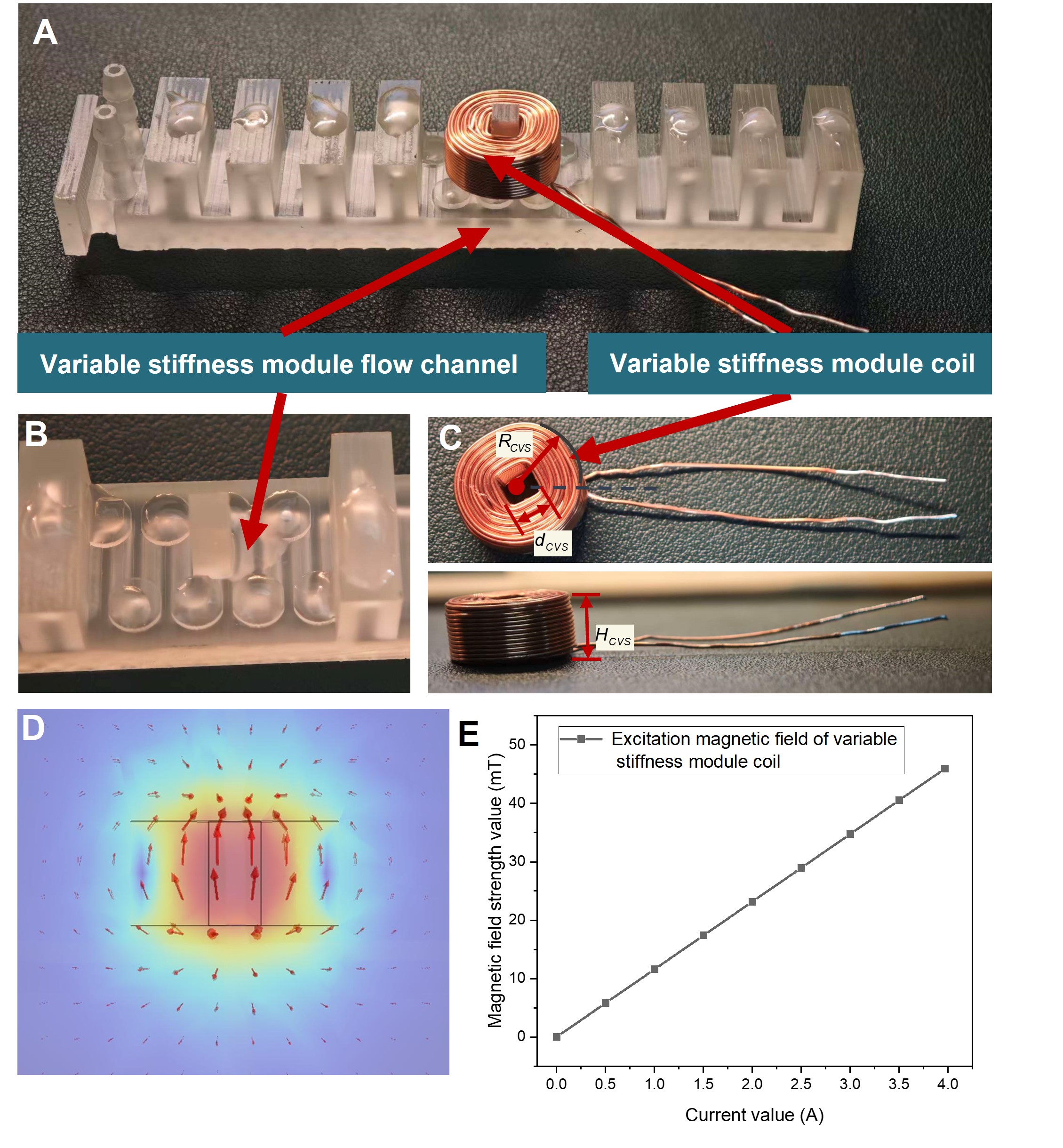 |
| --- |
| **FIGURE S5.** Variable stiffness module coil in MRF-VSSA. (A) Spatial relationship between the damping flow channel and coil in the variable stiffness module of MRF-VSSA. (B) Schematic diagram of the damping flow channel structure in the variable stiffness module. (C) Schematic diagram of key structural parameters of the coil. (D) Simulated magnetic field distribution of the coil. (E) Relationship curve between coil input current and response magnetic field. |

| 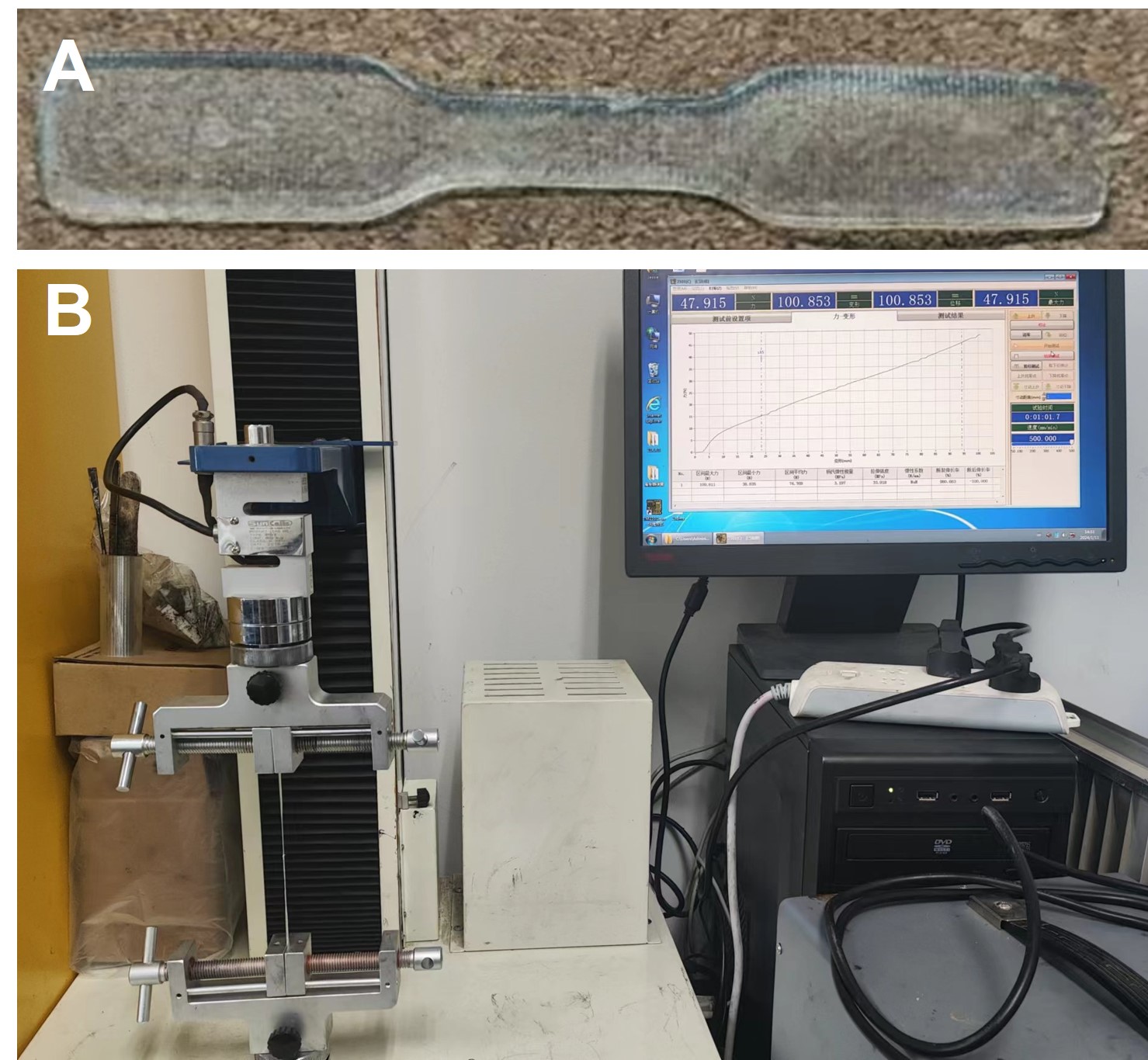 |
| --- |
| **Figure S6.** Tensile test of F39T flexible material. (A) Dumbbell-shaped Type 4 tensile specimen fabricated from F39T. (B) Tensile test of F39T specimen. |

| 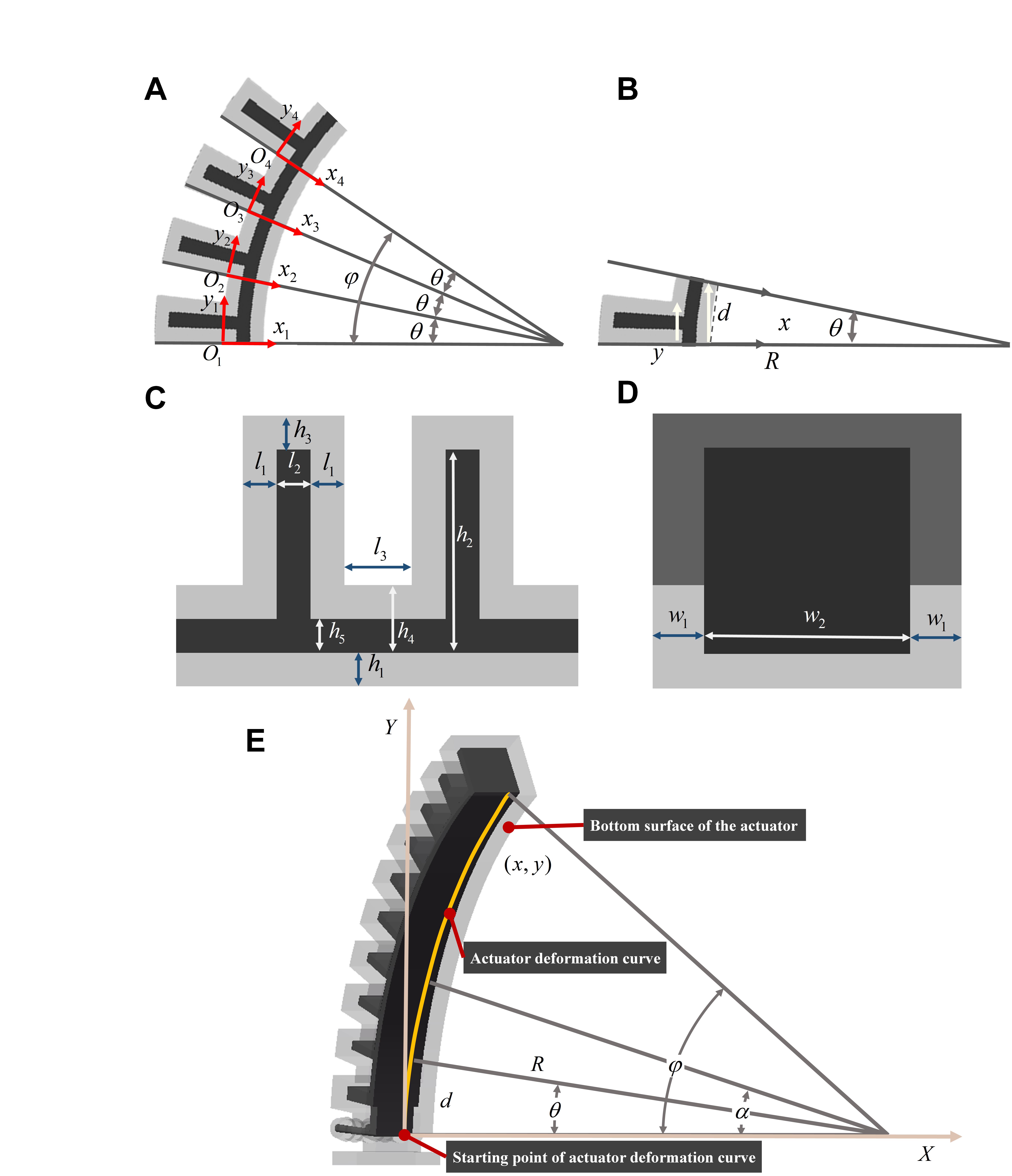 |
| --- |
| **FIGURE S7.** Schematic diagram of deformation analysis for MRF-SA. (A) Deformation analysis based on the segmented constant curvature assumption. (B) Bending deformation of a single chamber. (C) Parametric diagram of the rectangular chamber structure along the MRF-SA length direction. (D) Parametric diagram of the rectangular chamber structure along the MRF-SA width direction. (E) Bending deformation curve of the MRF-SA during predicted compression deformation. |

| 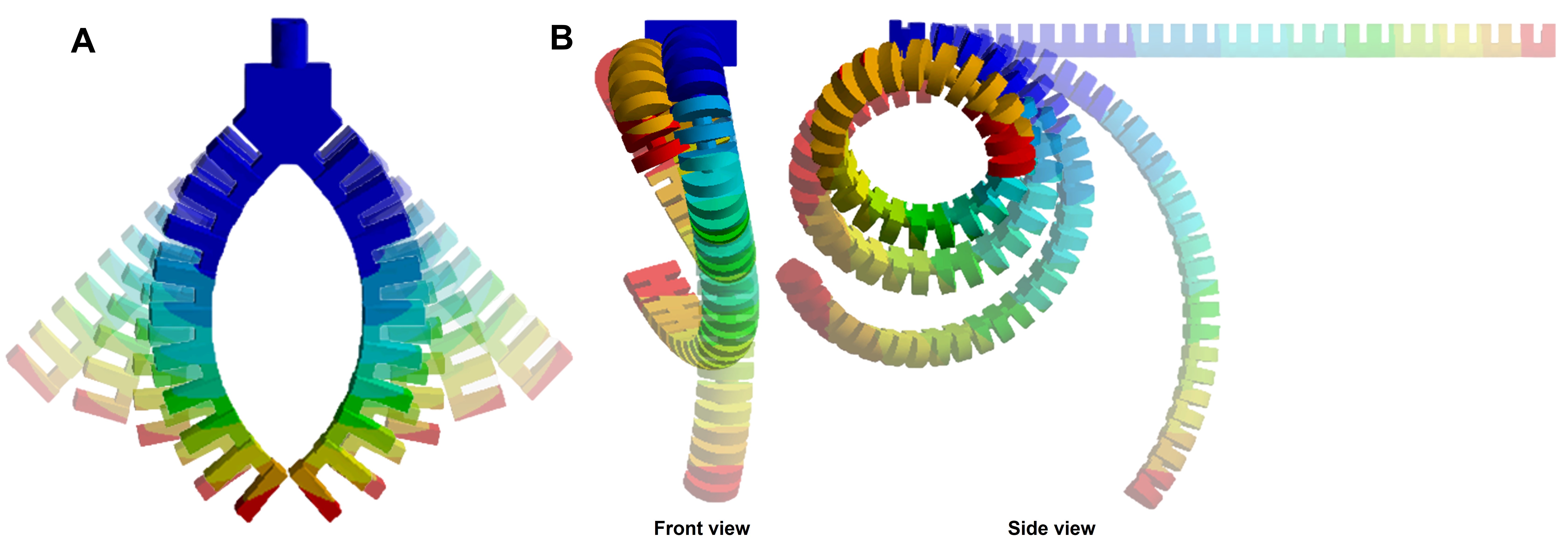 |
| --- |
| **FIGURE S8.** Deformation simulation of flexible grippers and soft trunk actuator. (A) The deformation of the internal cavity of the flexible gripper under pressure. (B) The deformation of the internal cavity of soft trunk actuator under pressure. |

| 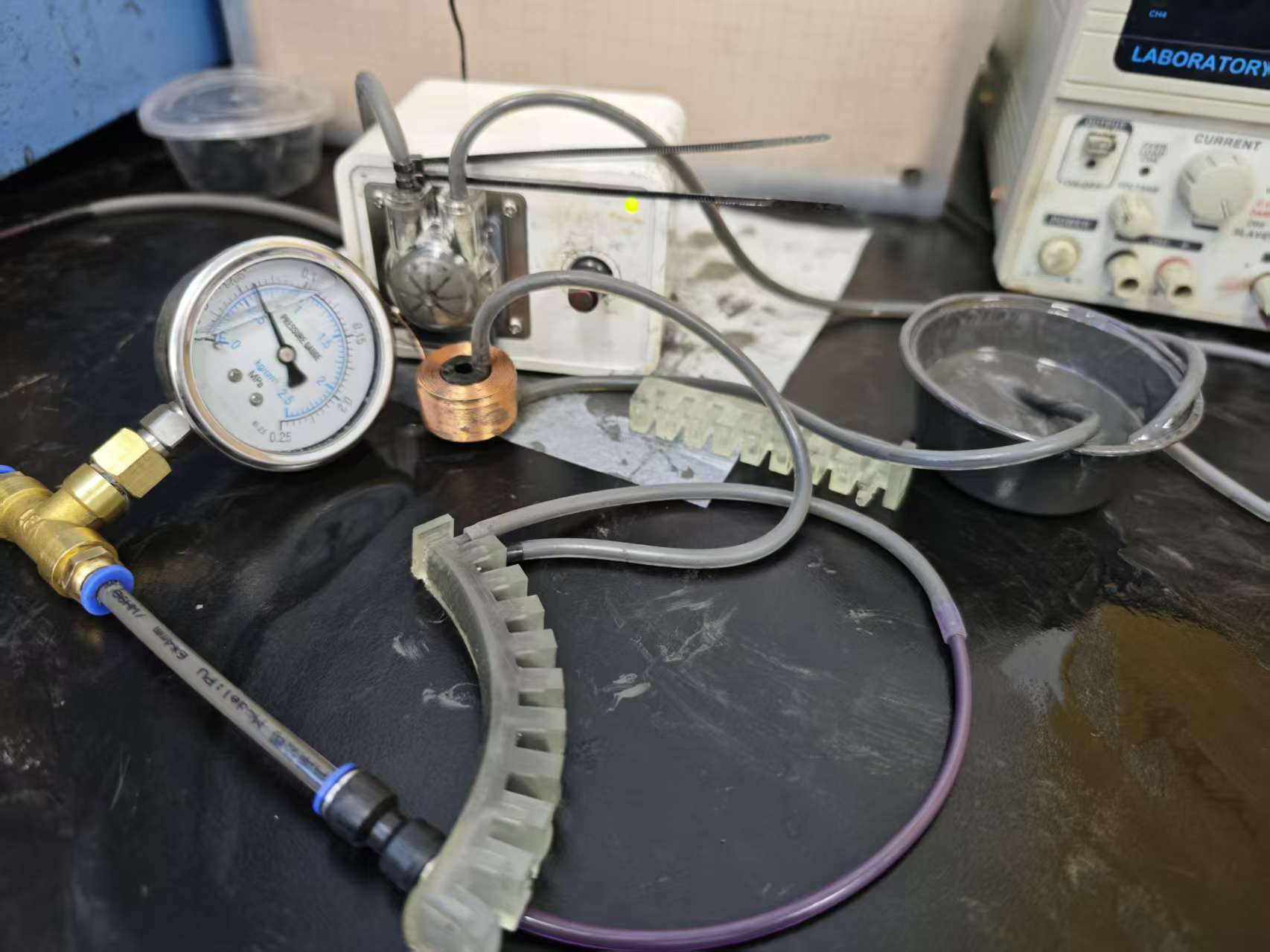 |
| --- |
| **FIGURE S9.** Back pressure in the test pipeline for bending deformation of soft actuators. |

| 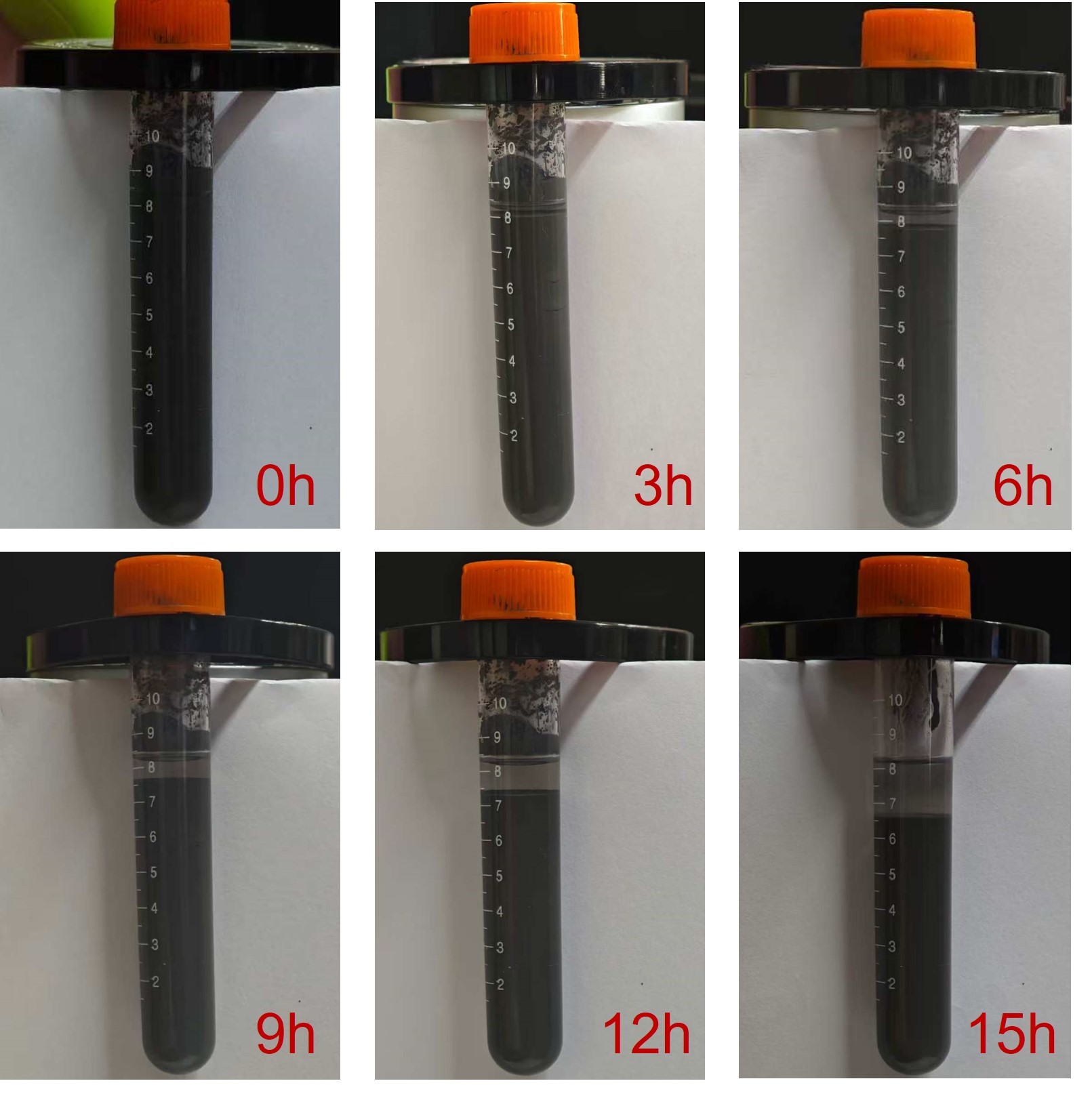 |
| --- |
| **FIGURE S10.** Experimental observation of the sedimentation characteristics of MRF-15D. |

| 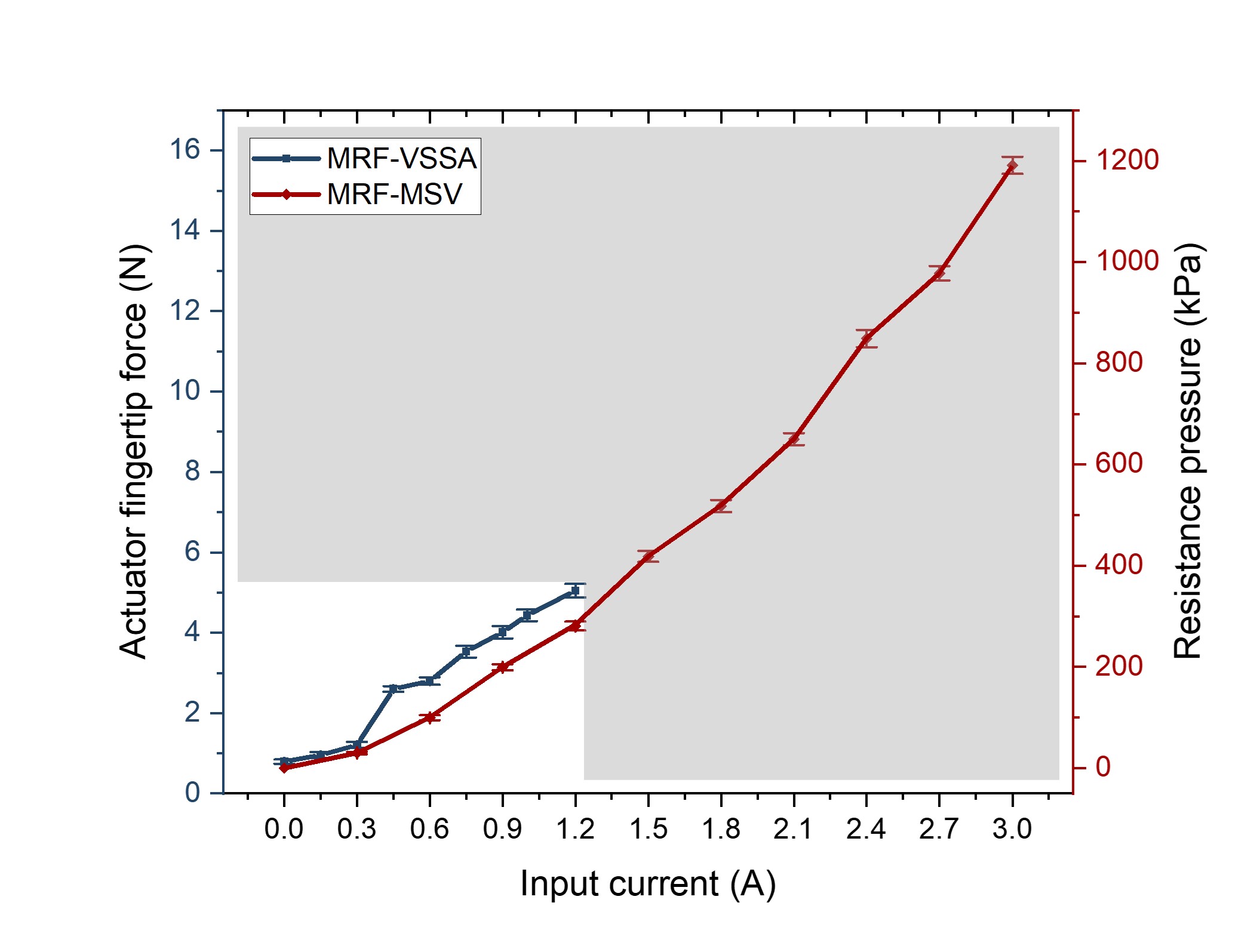 |
| --- |
| **FIGURE S11.** Relationship between MRF-MSV pressure drop and MRF-VSSA fingertip force as a function of input current. The error bars represent the standard deviation of the mean from five experimental trials. |

| 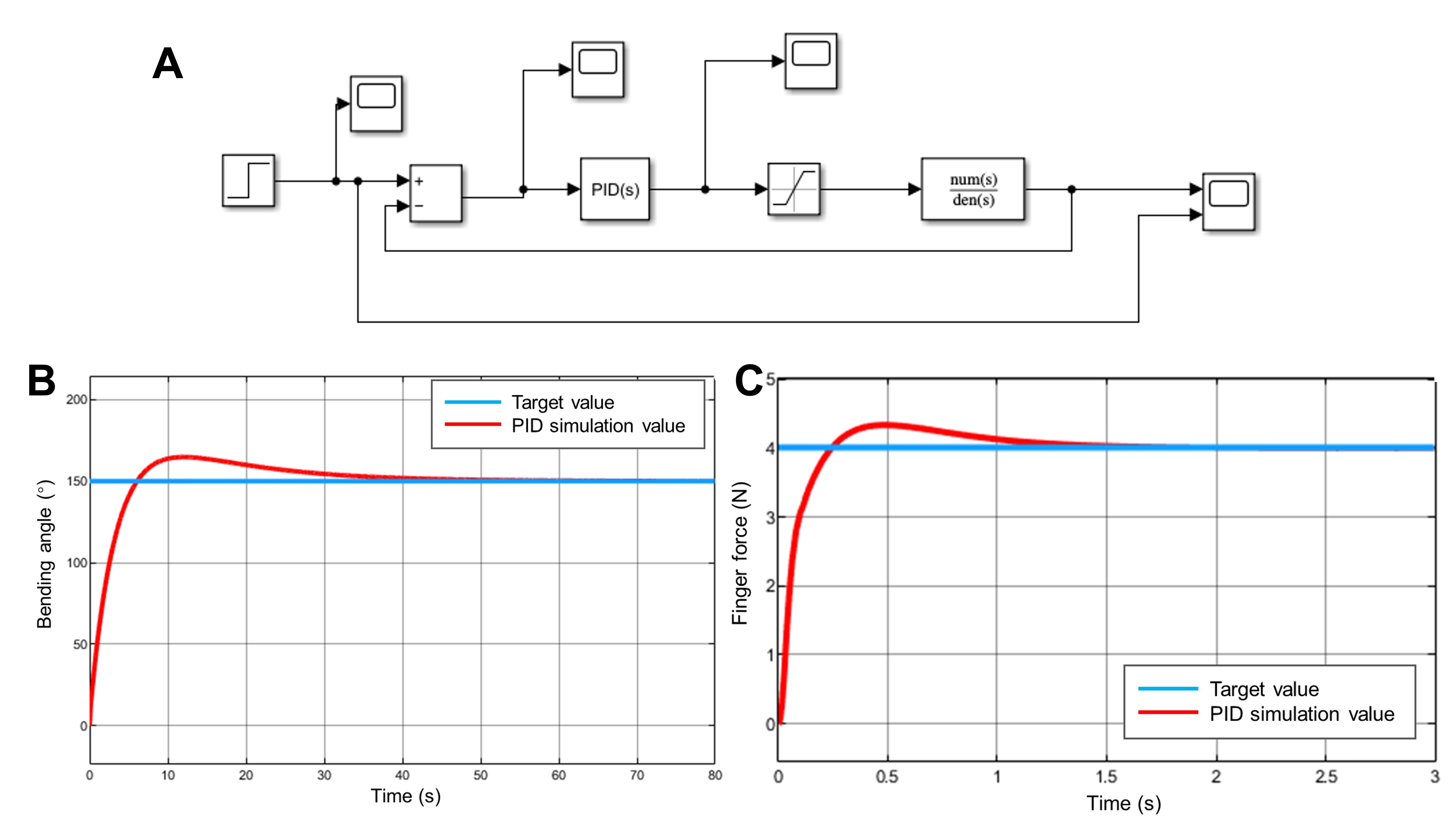 |
| --- |
| **FIGURE S12.** PID control simulation of the MRF-VSSA bending angle and fingertip force. (A) PID simulation control system; (B) Bending angle response, target value 150°; (C) Fingertip force response, target value 4 N. |

**TABLE S1. Yeoh third-order material modeling constants for F39T**

| **Photosensitive resin** | ***C_10_* (MPa)** | ***C_20_* (MPa)** | ***C_30_* (MPa)** |
| --- | --- | --- | --- |
| F39T | 1.242E-02 | -4.27E-08 | 8.24E-11 |

**TABLE S2. Simulation analysis of magnetorheological fluid viscosity changes upon entering smoothly damped flow channels at different pressures**

| **MRF pressure**  **(kPa)** | **Single layer damping channel** | **Cross-section of single-layer damping channel** |
| --- | --- | --- |
| 100 | 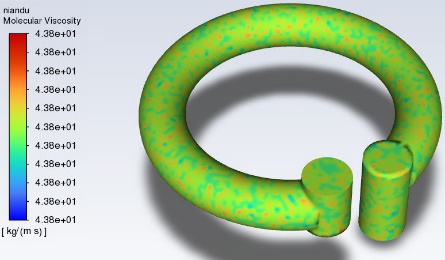 | 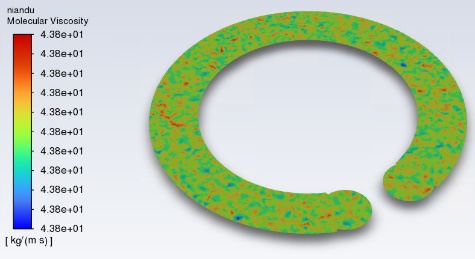 |
| 150 | 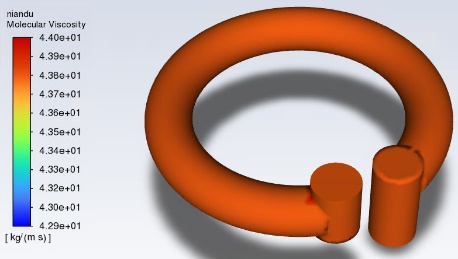 | 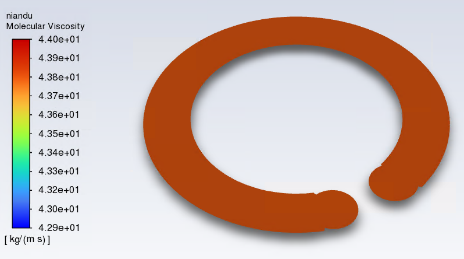 |
| 200 | 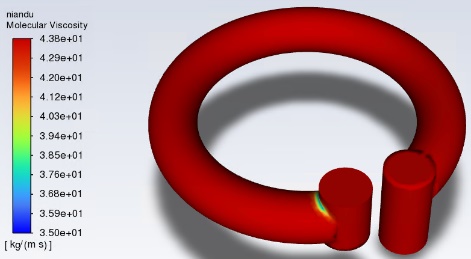 | 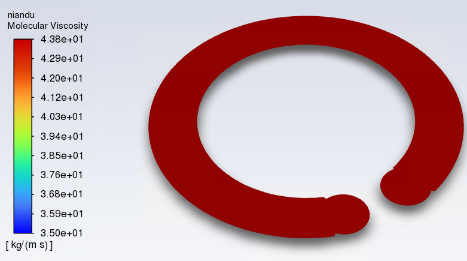 |
| 250 | 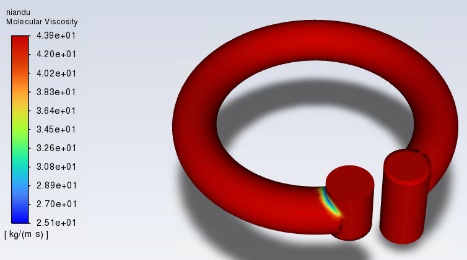 | 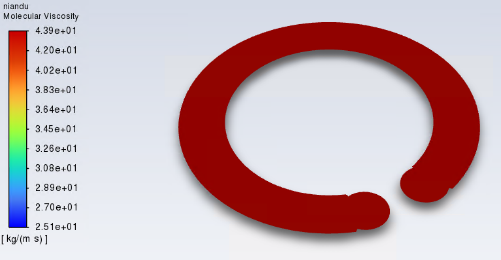 |
| 300 | 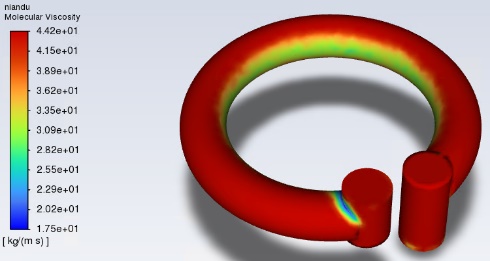 | 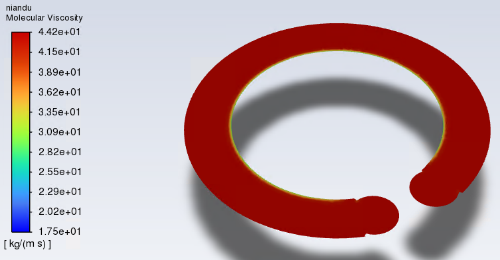 |
| 360 | 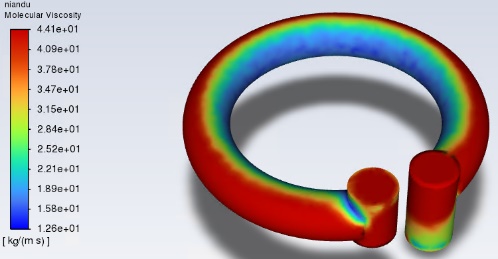 | 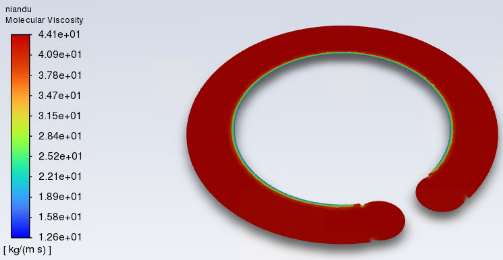 |
| 380 | 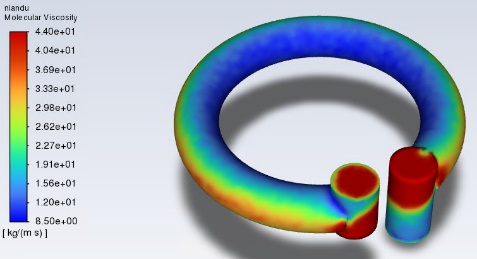 | 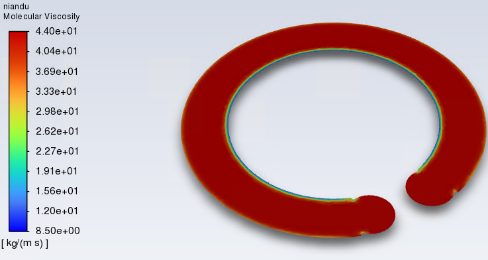 |
| 400 | 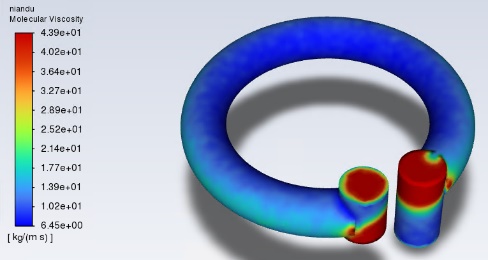 | 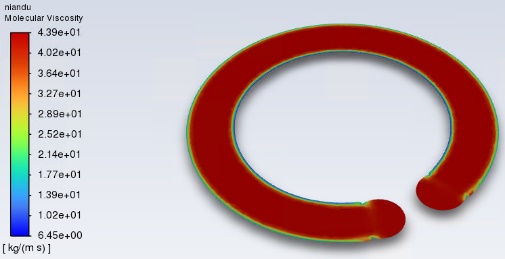 |

**TABLE S3. Simulation analysis of MRF viscosity changes upon entering a ring-shaped rectangular textured damping flow channel at different pressures**

| **MRF pressure**  **(kPa)** | **Single layer damping channel** | **Cross-section of single-layer damping channel** |
| --- | --- | --- |
| 100 | 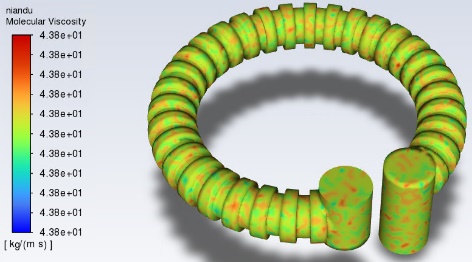 | 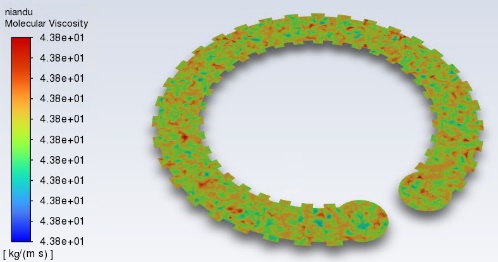 |
| 150 | 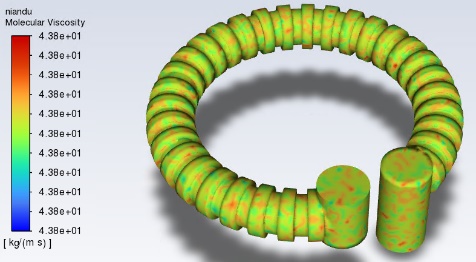 | 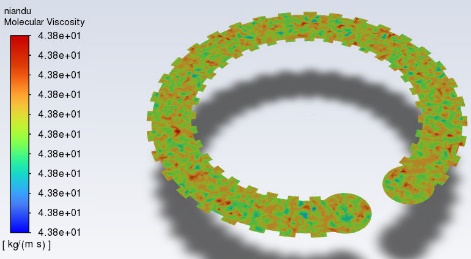 |
| 200 | 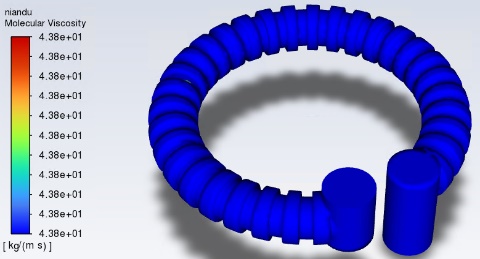 | 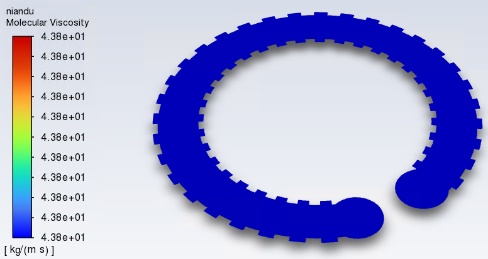 |
| 250 | 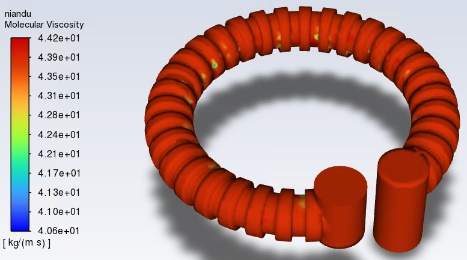 | 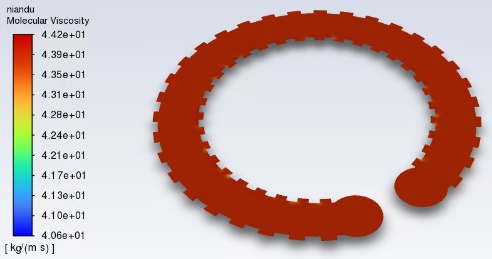 |
| 300 | 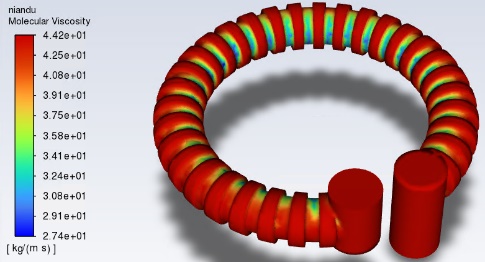 | 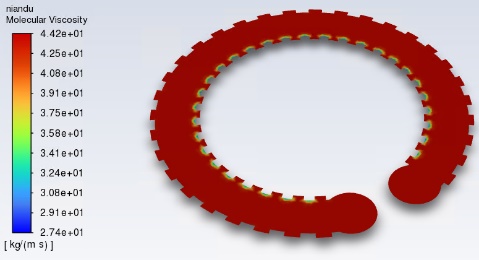 |
| 360 | 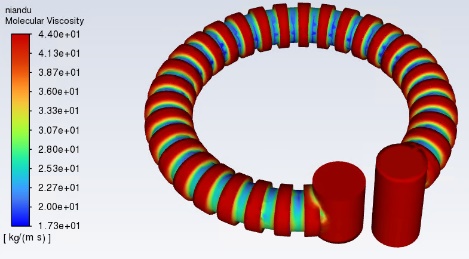 | 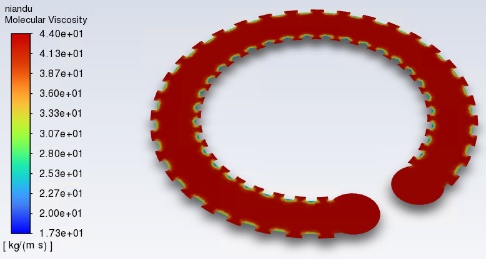 |
| 380 | 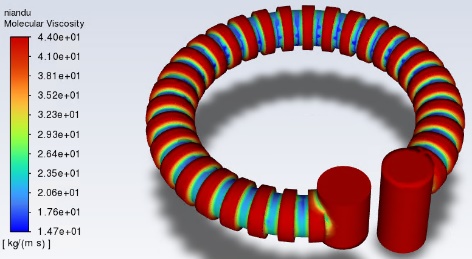 | 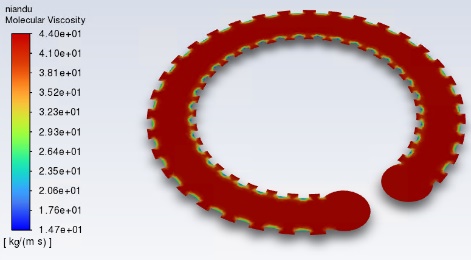 |
| 400 | 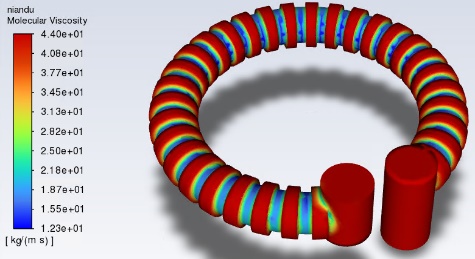 | 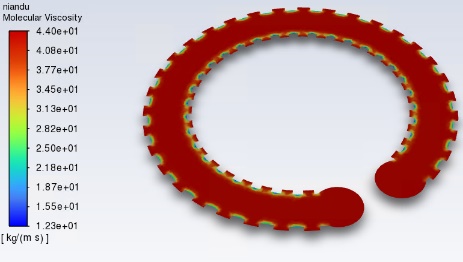 |

**Legends for supplementary videos**

**Movie S1:** Demonstration of the magnetic control resistance principle in MRF.

**Movie S2:** Action control test of magnetically controlled soft actuators.

**Movie** **S3:** Gesture action test of magnetically driven smart fluid manipulator.

**Movie** **S4:** Grasping test of magnetically driven smart fluid manipulator.
